# Registry‐Based Surveillance of Severe Acute Respiratory Infections in Norway During 2021–2024

**DOI:** 10.1111/irv.70080

**Published:** 2025-02-14

**Authors:** Elina Seppälä, Håkon Bøås, Jesper Dahl, Jeanette Stålcrantz, Melanie Stecher, Ragnhild Tønnessen, Gunnar Øyvind Isaksson Rø, Hilde Kløvstad, Trine Hessevik Paulsen

**Affiliations:** ^1^ Department of Infectious Disease Control and Vaccines Norwegian Institute of Public Health Oslo Norway; ^2^ ECDC Fellowship Programme, Field Epidemiology Path (EPIET) European Centre for Disease Prevention and Control (ECDC) Stockholm Sweden; ^3^ Department of Method Development and Analytics Norwegian Institute of Public Health Oslo Norway; ^4^ Department of Analysis and Diagnostics Norwegian Veterinary Institute Ås Norway

**Keywords:** COVID‐19, hospitalisation, influenza, registry‐based surveillance, RSV, severe acute respiratory infection (SARI)

## Abstract

**Background:**

In 2021, the Norwegian Institute of Public Health established temporary registry‐based surveillance of severe acute respiratory infections (SARI). We aimed to describe the surveillance system and evaluate selected attributes to inform the establishment of a permanent SARI surveillance system.

**Methods:**

SARI cases were defined using ICD‐10 discharge codes from national health and administrative registries, including codes for acute upper or lower respiratory infection (URI and LRI), COVID‐19, acute respiratory distress syndrome, pertussis or otitis media. Data from polymerase chain reaction (PCR) analyses were available for 10 respiratory pathogens including SARS‐CoV‐2, influenza virus and respiratory syncytial virus (RSV). We included data from 28 September 2020 to 31 March 2024 and calculated the following parameters: the proportion of cases tested for SARS‐CoV‐2, influenza virus and/or RSV; time between admission and registration of a SARI‐related ICD‐10 code; and proportion of cases with URI, LRI and COVID‐19.

**Results:**

We identified 214,730 SARI cases, of whom 82%, 73% and 53% were tested for SARS‐CoV‐2, influenza virus and RSV. Case peaks were predominantly driven by one or a combination of these pathogens. Median time between admission and a registered SARI diagnostic code was 5 (lower–upper quartile 3–10) days. Nowcasting and alternative case definitions for SARI with COVID‐19, influenza and RSV improved the timeliness. The ICD‐10 codes for LRIs and COVID‐19 captured only ~55% of the cases in the age group of 0–29 years compared to the routine case definition, where URIs were included.

**Conclusions:**

Registry‐based SARI surveillance provides timely data for handling epidemics of respiratory infections in Norway. We recommend establishing a permanent SARI surveillance system.

## 1 Introduction

Severe acute respiratory infections (SARI) continue to cause substantial morbidity and mortality worldwide, especially among young children and elderly individuals [1, 2]. The COVID‐19 pandemic and the co‐circulation of seasonal respiratory pathogens have strained healthcare systems, highlighting the importance of integrated SARI surveillance for preparedness, capacity planning and response [3]. Since 2020, the European Centre for Disease Prevention and Control (ECDC) has led several initiatives to implement and strengthen integrated SARI surveillance systems in Europe [4]. As part of these initiatives, many European countries have developed new hospital or registry‐based systems or strengthened existing systems [5, 6, 7].

Before the COVID‐19 pandemic, the surveillance of respiratory infections in Norway largely focused on influenza. In 2020, new indicator‐based surveillance systems, mostly registry based, were rapidly established in Norway to monitor COVID‐19 using the temporary Emergency preparedness register for COVID‐19 (Beredt C19) [8]. To prepare for the re‐emergence of seasonal respiratory infections, which had been absent during the first 1.5 years of the pandemic [9, 10], the Norwegian Institute of Public Health (NIPH) established a SARI surveillance system based on electronic health records (EHR) in Beredt C19 [4] and operated the system until Beredt C19 was closed on 30 June 2024. The aim of this paper is to describe this system and evaluate its timeliness, representativeness and the case definition used to provide recommendations for the establishment of a permanent SARI surveillance system in the future.

## 2 Methods

A temporary SARI surveillance system was established in the autumn of 2021 to monitor the seasonality, trends, severity and burden of severe respiratory infections to guide the response to the COVID‐19 pandemic. The system was national, comprehensive, passive and registry‐based and operated all year‐round. The catchment population comprised all residents, as well as visitors to Norway (population 5.6 million; see Supporting Information S1 for more demographic information).

### 2.1 Data Sources and Data Flow

**FIGURE 1:**
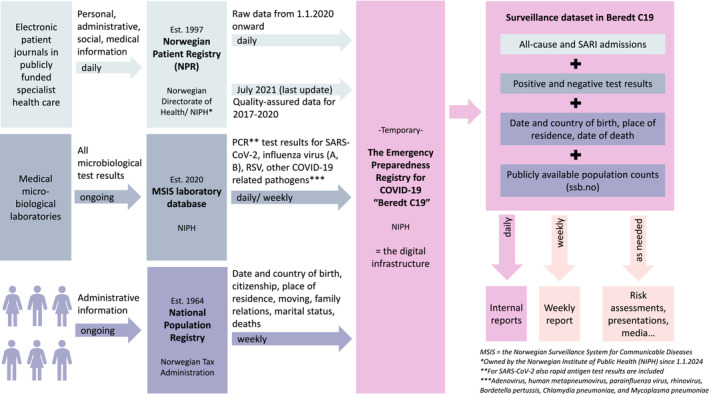
The key data sources and data flow for the Norwegian surveillance system for severe acute respiratory infections (SARI), 2021–2024.

The SARI surveillance system relied on individual‐level data from three key data sources: the Norwegian Patient Registry (NPR), which includes hospitalisation data from all publicly funded hospitals providing specialist healthcare in Norway [11]; the Norwegian Surveillance System for Communicable Diseases (MSIS) laboratory database, which includes data on positive and negative test results for SARS‐CoV‐2 and COVID‐19‐related pathogens (Supporting Information S2.2) from all microbiological laboratories in Norway [12]; and the National Population Registry, which includes administrative data on every individual who resides or has resided in Norway [13] (Figure 1). The core dataset could further be linked with supplementary data on, for example, vaccination against COVID‐19 and influenza from the Norwegian Immunisation Registry SYSVAK [14], and risk factors for severe COVID‐19 and influenza, which were defined based on data from NPR and the Norwegian Registry for Primary Health Care (NRPC) [15]. Descriptions of the Norwegian healthcare system and the key data sources are provided in Supporting Information S1 and S2.

Since April 2020, the individual‐level data were available for ongoing surveillance through Beredt C19, which was established according to the Act on Health and Social Preparedness §2–4 to provide NIPH an ongoing overview and knowledge of the prevalence, causal relationships and consequences of the COVID‐19 epidemic in Norway [8]. It served as the digital infrastructure, where raw data from the registries were updated, cleaned, linked, stored and analysed. Data linkage was based on pseudonymised unique identifiers and could be performed reliably for everyone who had a permanent Norwegian identification number (ID; assigned to everyone born in Norway and those granted residence permit (> 6 months) in Norway; stays unchanged throughout the individual's life). Data analyses were performed in Stata SE18 and R Version 4.3.2. Anonymised, aggregated results were published in weekly surveillance reports [10], risk assessments [16, 17] and presentations, and they were shared with the media and others requesting the data (Figure 1). The data were not reported to the European Surveillance System (TESSy), as further legal clarifications would have been needed to ensure that data could be shared internationally.

### 2.2 Case Definitions

The case definitions used are presented in Box 1. We selected a broad case definition comprising the 10th edition of the International Classification of Diseases (ICD‐10) diagnostic codes for acute upper and lower respiratory infections (URI and LRI; J00–J06 and J09–J22), acute respiratory distress syndrome in adults (ARDS; J80), COVID‐19 (U07.1 and U07.2), acute and unspecified otitis media (OM) (H65–H67) and pertussis (A37) (Table S1). These codes were selected based on consultation with experts at the NIPH, experiences from other countries and guidance from the Pan American Health Organization regarding active case finding of SARI cases in a sentinel surveillance system [18, 19, 20]. Both primary and secondary codes were included to determine the burden of SARI on hospitals. Furthermore, the hierarchy of coding follows specific national guidelines and is linked to funding, and thus, secondary codes may also contain information related to the main cause of admission.

Box 1 **Case definitions in the Norwegian surveillance system for severe acute respiratory infections (SARI), 2021–2024.**

**SARI case**: A hospitalised patient who has one or more of the ICD‐10 codes listed below as a primary or secondary diagnostic code registered at discharge and for whom the type of contact with specialist healthcare is registered as ‘admission’. Admissions registered ≥ 2 days apart are counted as new admissions. The ICD‐10 codes included in the surveillance are J00–J06 (acute upper respiratory infections), J09–J22 (acute lower respiratory infections including influenza and respiratory syncytial virus [RSV] infections), J80 (acute respiratory distress syndrome in adults), U07.1 and U07.2 (COVID‐19), A37 (pertussis) and H65–H67 (acute and unspecified otitis media only).

**Laboratory‐confirmed SARI case**: A SARI case who tested positive with PCR for at least one of the following respiratory pathogens: adenovirus (respiratory samples), human metapneumovirus, influenza virus (A and B), parainfluenza virus, rhinovirus, RSV, SARS‐CoV‐2 (PCR or antigen rapid test), *Bordetella pertussis* , *Chlamydia pneumoniae* , *Mycoplasma pneumoniae* , a maximum of 14 days before admission and up to 2 days after discharge.

**SARI‐COVID‐19 case**: A SARI case or a hospitalised patient who has not received any diagnostic code yet who tested positive for SARS‐CoV‐2 (PCR or antigen rapid test) a maximum of 14 days before admission and up to 2 days after discharge.

**SARI‐influenza case**: A SARI case or a hospitalised patient who has not received any diagnostic code yet who tested positive for influenza virus (A, B) a maximum of 14 days before admission and up to 2 days after discharge.

**SARI‐RSV case**: A SARI case or a hospitalised patient who has not received any diagnostic code yet who tested positive for RSV a maximum of 14 days before admission and up to 2 days after discharge.

**ICU SARI case**: A SARI case who received intensive care during the hospital stay. Information on intensive care is based on the Norwegian coding system for medical procedures (NCMP) and includes B0050. This procedure code was introduced in January 2022, and it has specific registration criteria*.

**SARI case with ventilatory support:** A SARI case who received invasive or non‐invasive ventilatory support during the hospital stay. The definition includes the NCMP codes GXAV01 (respirator treatment not further specified), GXAV10 (non‐invasive respirator treatment with continuous positive airway pressure; CPAP) and GXAV20 (non‐invasive respirator treatment with biphasic positive airway pressure; BiPAP).

**SARI death**: A SARI case who died during the hospital stay or within 14 days after discharge regardless of cause of death.

*https://www.helsedirektoratet.no/tema/finansiering/innsatsstyrt‐finansiering‐og‐drg‐systemet/innsatsstyrt‐finansiering‐isf

### 2.3 Descriptive Analysis of the Main Indicators

We described all SARI cases included in the SARI surveillance dataset (Figure 1) between Week 40 in 2020 (2020‐W40) and Week 13 in 2024 (2024‐W13) using the case definitions outlined in Box 1. We counted the cases by week or for the study period in total and by age group and sex. For SARI cases who had already been discharged, we also calculated the median (lower–upper quartile [LQ–UQ]) length of stay by age group. More results from the SARI surveillance system can be found in the weekly reports for COVID‐19 and other respiratory infections (in Norwegian) [10].

### 2.4 Evaluation of Selected Attributes

#### 2.4.1 Definition of SARI Based on ICD‐10 Codes

We compared the routinely used case definition including URI, LRI, COVID‐19, ARDS, pertussis and acute and unspecified OM with two alternative versions (URI, LRI and COVID‐19 and LRI and COVID‐19) to determine how much the different groups of diseases contributed to the overall burden of SARI. We also plotted the 3‐week moving average of number of hospital admissions by diagnostic code category (J00, J01, J02, etc.) to assess how these affected the burden of SARI.

As a secondary evaluation, we also compared several definitions of ‘hospitalised’, ‘laboratory‐confirmed’, ICU‐SARI and SARI deaths to the above mentioned routinely used case definitions, by using alternative cut offs of, for example, length of stay and time between hospitalisation and laboratory tests. The methods and results are discussed in Supporting Information S4 (including Tables S2–S5 and Figures S3–S10).

#### 2.4.2 Completeness

We assessed the completeness of the recorded age, sex and county of residence among the SARI cases by calculating the number and proportion of records with unknown or missing values. We also described the proportion of SARI cases who had a permanent Norwegian ID, which ensures that data in different registries can be reliably linked.

#### 2.4.3 Testing Practices and Representativeness of Testing

Up until April 2022, it was recommended that everyone with acute respiratory symptoms should be tested for SARS‐CoV‐2, either by PCR or by self‐test, the results of which were to be confirmed by PCR up to January 2022 [21]. Testing outside of this is primarily based on clinical decision. Hence, testing practices may vary by age group and geographical area. We excluded cases without a permanent Norwegian ID and described the PCR testing practices for SARS‐CoV‐2, influenza virus and RSV by calculating the proportion of SARI cases who were tested for one, two or all three of these pathogens on different days or the same day. We also assessed the representativeness of the data for SARI cases tested and by calculating the proportion of cases tested and the number of positive results for each of these pathogens by age group and county.

#### 2.4.4 Timeliness

Basing the SARI surveillance system on discharge codes makes timeliness a challenge. Nowcasting has therefore been routinely applied to estimate the number of SARI cases for the weeks assumed to be incomplete (Supporting Information S5, including Figure S11). NPR does not contain time stamps for when data for patients are registered or updated. Therefore, to assess the timeliness of the surveillance system for this study, we established a system that artificially created registration dates for prespecified data items in the surveillance dataset by comparing the newest dataset to the dataset from the previous day. We collected data through this system between 18 December 2023 and 10 March 2024 and extracted data on SARI cases with complete records to calculate the median number of days (LQ–UQ) for the following indicators:

- admission—patient visible in the dataset with or without diagnostic code
- admission—patient visible in the dataset with a SARI code
- start of procedure—procedure code visible in the dataset
- death—death visible in the dataset and
- date of testing for SARS‐CoV‐2, influenza virus and/or RSV—result visible in the dataset.

Whereas discharge codes are typically registered at the time of discharge, diagnostic codes may sometimes be recorded as soon as the diagnosis is confirmed, even though the patient is still hospitalised. We calculated the proportion of SARI cases who had a SARI code registered before their final discharge date from the hospital.

### 2.5 Ethical Considerations and Data Protection

The Act on Health and Social Preparedness §2–4 gave the NIPH a legal mandate to use and link registry data and thus perform surveillance of COVID‐19 and related infectious diseases without informed consent or need for ethical approval during the COVID‐19 pandemic. All analyses performed inside Beredt C19 complied with the data protection impact assessment (DPIA) of Beredt C19. National IDs were pseudonymised with an algorithm recommended by the National Security Authority so that only a non‐reversible identifier was used when linking personal information from various sources. Only anonymous, aggregated results were exported from Beredt C19.

## 3 Results

### 3.1 Description of the SARI Cases

**FIGURE 2:**
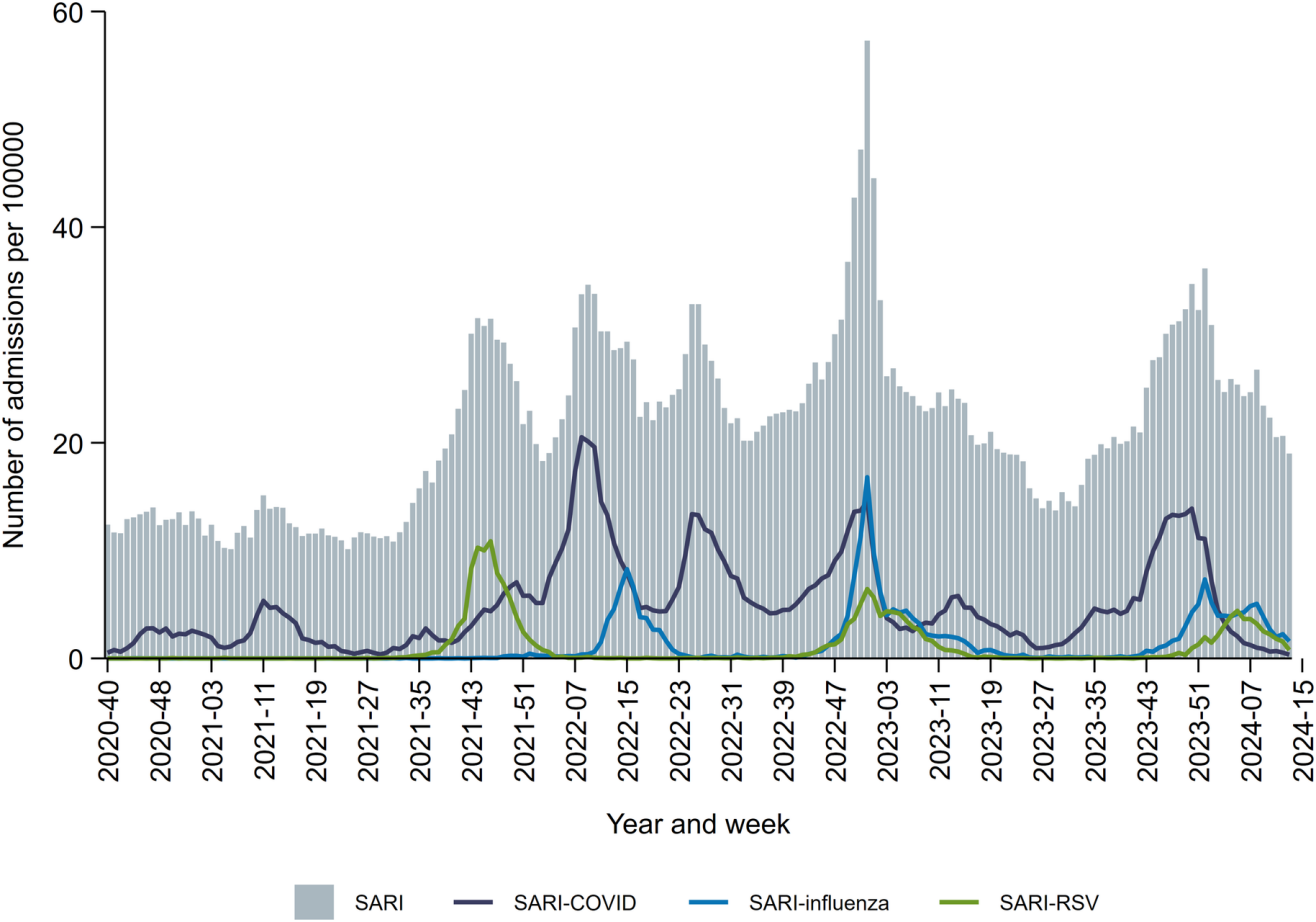
Weekly number of admissions per 100,000 with severe acute respiratory infection (SARI), SARI‐COVID‐19, SARI‐influenza and SARI‐RSV, Norway, 28 September 2020–31 March 2024.

Between 2020‐W40 and 2024‐W13, 214,730 SARI cases were reported, comprising 7% of all admissions (*N* = 3,002,560) in publicly funded specialist healthcare in Norway during this period. Of these, 23% (49,736) tested positive for SARS‐CoV‐2, 6% (12,903) for influenza virus and 5% (10,300) for RSV. Several peaks of SARI admissions were observed, predominantly driven by one or a combination of these pathogens (Figure 2). The median age at admission was 71 years (LQ‐UQ 46–81 years), and 46% (97,906) were female. The median length of stay was 4 (LQ‐UQ 2–7) days. The cases in the age groups 0–4 and 5–14 years had the shortest length of stay with 1 (LQ‐UQ 1–3) day, and 11% (2619/23,685) and 12% (595/4983) of the admissions in these age groups were shorter than 12 h (Table S2). The elderly had the longest median length of stay: 5 (LQ‐UQ 3–9) days for 65‐ to 79‐year‐olds, and 5 (LQ‐UQ 3–8) days for the ≥ 80‐year‐olds. Of all cases, 10% (20,507) received invasive and/or non‐invasive ventilatory support, and 5% (10,491) received intensive care with or without ventilatory support. Eight per cent (16,835) died in hospital or within 14 days after discharge, primarily among the oldest age groups (data not shown).

### 3.2 Evaluation of Selected Attributes

#### 3.2.1 Definition of SARI Based on ICD‐10 Codes

When comparing different SARI case definitions based on ICD‐10 codes, we found that excluding diagnostic codes for pertussis, OM and ARDS led to a 1.5% (3267/214,501) decrease in the number of cases (Table 1). Excluding the diagnostic codes for URIs in addition to these led to a 42%–47% (4535/10,715; 2298/4962) reduction in the number of captured cases among 0‐ to 29‐year‐olds compared to the broadest case definition. Figure S1 compares the three case definitions over time, and Figure S2 shows the 3‐week moving average of the number of hospital admissions by diagnostic code category.

**TABLE 1:** Number of admissions with severe acute respiratory infection (SARI) by age group and case definition, Norway, 28 September 2020–31 March 2024.

	Case definition				
	1 URI^a^, LRI^b^, COVID‐19^c^, ARDS^d^, pertussis^e^, OM^f^	2 URI^a^, LRI^b^, COVID‐19^c^		3 LRI^b^, COVID‐19^c^	
Age group	*n*	*n*	% of case definition 1	*n*	% of case definition 1
0–4 years	23,647	22,438	94.9	13,339	56.4
5–14 years	4926	4649	94.4	2628	53.3
15–29 years	10,715	10,510	98.1	6181	57.7
30–64 years	46,835	46,001	98.2	40,475	86.4
65–79 years	68,830	68,277	99.2	65,383	95.0
80+ years	59,548	59,350	99.7	57,314	96.2
Total	214,501	211,225	98.5	185,320	86.4

Abbreviations: ARDS = acute respiratory distress syndrome in adults, LRI = lower respiratory infection, OM = acute and unspecified otitis media, URI = upper respiratory infection.

^a^ URI: J00–J06.

^b^ LRI: J09–J22.

^c^ COVID‐19: U07.1 and U07.2.

^d^ ARDS: J80.

^e^ Pertussis: A37.

^f^ OM: H65–H67.

#### 3.2.2 Completeness

All 214,730 cases had information on age, and all except two had information on sex. The county of residence was known for 99% (212,270) of the cases, and less than 1% of the SARI cases occurred among tourists and others without long‐term residency in Norway.

#### 3.2.3 Testing Practices and Representativeness of Testing

Approximately 52% of the SARI cases included in this analysis (*N* = 212,933) were tested for SARS‐CoV‐2, influenza virus and RSV; the majority were tested on the same day (Table 2). The testing practices varied between age groups: 79% and 68% of the SARI cases in the 0–4 years (*N* = 23,558) and 5–14 years (*N* = 4894) age groups, respectively, were tested for all three pathogens, but testing for RSV in addition to SARS‐CoV‐2 and influenza virus was less common in the other age groups (Table 2). Of the 212,933 SARI cases, 82%, 73% and 53% were tested for SARS‐CoV‐2, influenza virus and RSV, respectively. The proportion tested varied with age group (77%–87% for SARS‐CoV‐2, 65%–85% for influenza and 47%–80% for RSV) (Table S7). The testing practices also varied between counties, with the proportions of cases tested for SARS‐CoV‐2, influenza virus and RSV ranging from 57% to 91%, 45% to 85% and 14% to 76%, respectively (Table S8).

**TABLE 2:** Number and proportion of admissions with severe acute respiratory infection (SARI) by testing strategy for SARS‐CoV‐2, influenza virus and/or respiratory syncytial virus (RSV) and age group, Norway, 28 September 2020–31 March 2024. Only SARI cases with a permanent Norwegian ID were included.

Testing strategy		Total		0–4 years		5–14 years		15–29 years		30–64 years		65–79 years		80+ years	
		*n*	%	*n*	%	*n*	%	*n*	%	*n*	%	*n*	%	*n*	%
Not tested		35,923	16.9	2938	12.5	970	19.8	2324	22.0	7388	16.0	11,366	16.6	10,937	18.4
Only SARS‐CoV‐2		20,737	9.7	547	2.3	208	4.3	1332	12.6	6727	14.6	6510	9.5	5413	9.1
Only influenza virus		730	0.3	10	0.0	5	0.1	27	0.3	157	0.3	246	0.4	285	0.5
Only RSV		96	0.0	52	0.2	2	0.0	2	0.0	10	0.0	22	0.0	8	0.0
Only SARS‐CoV‐2 and influenza virus															
	Different days	1437	0.7	32	0.1	9	0.2	129	1.2	486	1.1	446	0.7	335	0.6
	Same day	41,845	19.7	1135	4.8	328	6.7	1846	17.4	8798	19.1	15,370	22.5	14,368	24.2
Only SARS‐CoV‐2 and RSV															
	Different days	140	0.1	4	0.0	0	0.0	7	0.1	46	0.1	48	0.1	35	0.1
	Same day	192	0.1	37	0.2	3	0.1	1	0.0	42	0.1	52	0.1	57	0.1
Only influenza virus and RSV															
	Different days	4	0.0	0	0.0	0	0.0	0	0.0	3	0.0	0	0.0	1	0.0
	Same day	934	0.4	132	0.6	25	0.5	36	0.3	178	0.4	275	0.4	288	0.5
SARS‐CoV‐2, influenza virus and RSV															
	Different days	10,265	4.8	584	2.5	121	2.5	543	5.1	3017	6.5	3605	5.3	2395	4.0
	Same day	100,630	47.3	18,087	76.8	3223	65.9	4337	41.0	19,313	41.8	30,455	44.5	25,215	42.5
Total		212,933	100.0	23,558	100.0	4894	100.0	10,584	100.0	46,165	100.0	68,395	100.0	59,337	100.0

#### 3.2.4 Timeliness

To evaluate the timeliness of the surveillance, we included 17,879 SARI cases admitted between 18 December 2023 and 10 March 2024. The median time between admission and registration of the admission was 1 day, but data on PCR test results were available for 50% of the cases within 2 days after sampling (Table 3). The time between admission and registration of a diagnostic code for SARI was 5 days, and 67% of the SARI cases received their diagnosis within one week after admission. Shorter length of stay corresponded with less time between admission and registration of diagnostic code(s), resulting in better timeliness for younger age groups (data not shown). Of all cases, 25% received a diagnostic code for SARI before the final discharge date from the hospital. The technical difficulties that some health trusts had in submitting data to NPR during the data collection period did not affect the median number of days between events and registration (data not shown). The longest delay between event and data being available in the dataset was observed for information on deaths and intensive care (Table 3).

**TABLE 3:** Time in days between the event and the data being available in the surveillance database for severe acute respiratory infection (SARI), Norway, 18 December 2023–March 31 2024.

		Time in days		
Indicator	*N*	Median	Lower quartile	Upper quartile
Admission—patient visible in the surveillance database with or without dg code	17,871	1	1	2
Admission—patient visible in the surveillance database with SARI code	17,871	5	3	10
Date of testing—result available in the surveillance dataset	14,851	2	1	5
Start of intensive care (IC)—procedure code for IC visible in the surveillance database	1942	6	2	12
Death—death visible in the surveillance database	1209	3	1	7

## 4 Discussion

We successfully established a temporary registry‐based SARI surveillance system utilising a new digital infrastructure in Norway [8]. With high data completeness, the surveillance system captured several peaks of respiratory infections, which were driven by COVID‐19, influenza and/or RSV. These findings are in line with surveillance data from other Norwegian surveillance systems [10]. Even though the results were not reported to TESSy due to unclarities regarding data sharing, they were regularly communicated to the Norwegian public and stakeholders to inform control measures and capacity planning in the healthcare sector [16, 17]. Alongside frequent data updates, having access to individual‐level data with the possibility of linking data between registries was one of the most significant strengths of the system, making it possible for the SARI surveillance team to quickly respond to changing needs.

When the system was established, there were no recommendations available from the WHO European Region or ECDC regarding which ICD‐10 codes to include in the case definition of SARI in a registry‐based surveillance system. Symptom‐based case definitions cannot usually be applied to registry‐based surveillance systems relying on discharge codes, and countries with registry‐based SARI surveillance systems have used varying case definitions based on local coding practices and needs [6, 18, 22, 23]. When establishing the system, we chose a highly sensitive case definition by including URIs and both primary and secondary diagnostic codes and by defining a new admission as any admission separated by more than 1 day from the last discharge date. Hence, we prioritised the monitoring of the burden of SARI on specialist healthcare over monitoring the severity of epidemics. We found that although the inclusion of diagnostic codes for pertussis and OM contributed very little to the number of identified cases in most age groups, a significant number of SARI cases among children and young adults would have been missed if URIs had been excluded. Although the trends were very similar for the different combinations of ICD‐10 codes we studied, case definitions that exclude URIs may underestimate the burden of SARI on the healthcare system, especially pediatric services. As a precautionary principle, the threshold for admitting infants is generally lower than for other age groups. However, being hospitalised in Norway indicates a certain degree of severity, as primary care physicians function as gatekeepers for specialised services, and health services outside of hospitals are readily accessible. Therefore, including both URIs and LRIs in the case definition in the Norwegian context captures the burden of respiratory infections in both the pediatric population and pediatric secondary healthcare services.

Diagnostic codes for chronic respiratory diseases were excluded from the system. This may have caused the system to miss some acute lower respiratory infections related to chronic pulmonary diseases. Therefore, codes such as J44.0 (chronic obstructive pulmonary disease with acute lower respiratory infection) should be considered for inclusion in the future, as some of these codes have frequently been registered for patients admitted with COVID‐19 as main cause in Norway [24]. It should also be evaluated whether tuberculosis and legionellosis, where transmission is somewhat different to other respiratory pathogens, should be included.

In addition to differences in diagnostic codes included in SARI case definitions, there is large heterogeneity in the existing registry‐based SARI surveillance systems in European countries with regard to the identification of hospitalised patients, severity indicators and availability of microbiological results [6, 18, 22, 23]. Where data on microbiological results are available, testing practices can vary. Apart from SARS‐CoV‐2, there have not been national guidelines for testing patients with SARI for respiratory pathogens, and testing practices can be influenced by the expected epidemiological situation, reimbursement, practical arrangements and public attention, among others. Understanding testing practices and the representativeness of testing is important for interpreting the results from the SARI surveillance. Testing for SARS‐CoV‐2 was more common than testing for influenza virus and RSV, with the highest testing activity observed among the age group 0–4 years for all three pathogens. Interestingly, < 50% of the individuals in the 65+ age group were tested for RSV, even though RSV is known for its ability to cause severe disease among the elderly in addition to young children [25]. The testing activity varied greatly between counties, suggesting that some hospitals may use antigen rapid tests or other point of care tests, the results of which are not reported in the MSIS laboratory database. Some age groups and counties may thus be under‐represented when monitoring SARI‐COVID‐19, SARI‐influenza and SARI‐RSV.

Even with daily updated data on hospitalisations, identifying SARI cases based on discharge codes inevitably challenges the timeliness of the surveillance system. We found that > 30% of the SARI cases were not detected until more than one week after admission. Two different methods were applied to increase timeliness. Having access to positive test results for SARS‐CoV‐2, influenza virus and RSV, which were updated weekly or daily, enabled us to compensate for this delay when monitoring SARI‐COVID‐19, SARI‐influenza and SARI‐RSV. In these case definitions, hospitalised patients with laboratory confirmation, but no diagnostic code yet, were included. Using this method allowed the surveillance system to detect these cases earlier, initially also including some patients that later were excluded as they were discharged without a SARI code. In addition, we applied nowcasting [25], which was especially useful for admissions with diagnostic codes that did not have a corresponding microbiological confirmation, such as bacterial pneumonia. If provisional diagnostic codes were already registered at admission, the timeliness of the system could likely be further improved.

Another strength of the surveillance system was including other respiratory pathogens in addition to SARS‐CoV‐2, influenza virus and RSV, which enabled the identification and monitoring of smaller epidemics of, for example, hMPV, *Bordetella pertussis* and *Mycoplasma pneumoniae* [10], and the investigation of some signals related to selected respiratory pathogens detected in other countries. Including microbiological data on other respiratory pathogens such as other human coronaviruses, enteroviruses, human parechovirus, bocavirus, *Streptococcus pneumoniae* and *Streptococcus pyogenes* should be considered to obtain an even better overview of the epidemiology of respiratory infections in Norway. However, the current legislation allows the storage of directly identifiable data only for selected pathogens, and the possibilities for linkage of registries are therefore currently limited. Having access to historical data from NPR was important because it provided some context to changes seen in the epidemiology of seasonal respiratory infections during the COVID‐19 pandemic. Three more years of historical data could have enabled the definition of epidemic thresholds using, for example, the moving epidemic method (MEM) [26], emphasising the point that sufficient historical data should be available when establishing a new surveillance system or an emergency preparedness registry.

In addition to the challenges of timeliness and representativeness of testing, the surveillance system also had some other limitations. It was not possible to reliably determine whether patients were admitted due to or with SARI. For monitoring the burden of SARI on hospitals, this distinction is less relevant, as both patient groups likely require infection control measures, but, for example, in vaccine effectiveness analyses, it may be desirable to include only patients severely ill due to SARI to improve precision [27]. Furthermore, the elderly may have been somewhat under‐represented in the surveillance due to alternative arrangements in primary care for treating this patient group.

Perhaps the most important limitation of the SARI surveillance system, however, was its temporary nature. The Act on Health and Social Preparedness §2–4 gave the NIPH the legal right to establish Beredt C19 and to collect and link individual‐level data from national registries needed for controlling the COVID‐19 pandemic. According to this act, Beredt C19 was to be closed and the data to be deleted as soon as the pandemic was over and evaluated. The NIPH is working on establishing new permanent solutions for registry‐based surveillance of infectious diseases, including SARI, through multiple projects.

## 5 Conclusion

The temporary SARI surveillance system relying on data linkage through unique personal identifiers was a proof of concept demonstrating that registry‐based surveillance can supply timely and complete surveillance data for handling epidemics of respiratory infections in Norway. We recommend establishing a permanent registry‐based SARI surveillance system. Having access to provisional diagnostic codes and linkable data on a wider range of respiratory pathogens would increase the value of the surveillance system, including for preparedness purposes.

## Author Contributions

**Elina Seppälä:** conceptualization, methodology, visualization, writing – original draft, formal analysis. **Håkon Bøås:** conceptualization, methodology, writing – review and editing. **Jesper Dahl:** conceptualization, methodology, writing – review and editing. **Jeanette Stålcrantz:** writing – review and editing. **Melanie Stecher:** writing – review and editing. **Ragnhild Tønnessen:** writing – original draft, writing – review and editing, conceptualization. **Gunnar Øyvind Isaksson Rø:** conceptualization, methodology, formal analysis, writing – original draft, writing – review and editing, visualization. **Hilde Kløvstad:** conceptualization, supervision, writing – review and editing. **Trine Hessevik Paulsen:** conceptualization, methodology, supervision, project administration, writing – review and editing.

## Ethics Statement

The Act on Health and Social Preparedness §2–4 gave the Norwegian Institute of Public Health a legal mandate to use and link registry data and thus perform surveillance of COVID‐19 and related infectious diseases without informed consent or need for ethical approval during the COVID‐19 pandemic.

## Conflicts of Interest

The authors declare no conflicts of interest.

### Peer Review

The peer review history for this article is available at https://www.webofscience.com/api/gateway/wos/peer‐review/10.1111/irv.70080.

## Supporting information


**Table S1** International Classification of Diseases (ICD)‐10 codes in the Norwegian patient registry (NPR) included in the routine surveillance of severe acute respiratory infections (SARI), Norway, 2021–2023.
**Table S2.** Length of stay for admissions with severe acute respiratory infection (SARI) by age group, Norway, 28 September 2020–31 March 2024. LQ = lower quartile, UQ = upper quartile.
**Table S3.** Number and proportion of admissions linking to hospital admissions by type of admission (all‐cause and SARI), pathogen, test result and timing of test, Norway, 28 September 2020–31 March 2024.
**Table S4.** Number and proportion of admissions with severe acute respiratory infection (SARI) where the patient was tested and tested positive for SARS‐CoV‐2, influenza virus and respiratory syncytial virus (RSV), by definition for ‘tested’, Norway, 28 September 2020–31 March 2024 (total number of admissions with SARI among patients with a permanent Norwegian ID = 212,933).
**Table S5.** Sensitivity, specificity and positive and negative predictive value (PPV, NPV) of different case definitions for intensive care admissions with severe acute respiratory infection (SARI) and positive PCR for SARS‐CoV‐2 (SARI‐COVID) or for influenza virus (SARI‐influenza), compared to ICU admissions with confirmed COVID‐19 or influenza registered in the Norwegian Intensive Care Registry (NIR, N_COVID‐19_ = 1530, N_influenza_ = 416), Norway, 3 January 2022–31 March 2024.
**Table S6.** Number and proportion of deaths associated with admission with severe acute respiratory infection (SARI) where a diagnostic code for SARI was registered in the death certificate, by timing of death in relation to the hospital stay, Norway, 28 September 2020–31 March 2024.
**Table S7.** Number and proportion of admissions with severe acute respiratory infection (SARI) where the patient was tested for SARS‐CoV‐2, influenza virus and respiratory syncytial virus (RSV) by age group, Norway, 28 September 2020–31 March 2024. Only SARI cases with a Norwegian ID are included.
**Table S8.** Number and proportion of admissions with severe acute respiratory infection (SARI) where the patient was tested for SARS‐CoV‐2, influenza virus and respiratory syncytial virus (RSV) by county, Norway, 28 September 2020–31 March 2024. Only SARI cases with a Norwegian ID are included.
**Figure S1.** Weekly number of admissions with severe acute respiratory infection (SARI) by case definition, Norway, 28 September 2020–31 March 2024.
**Figure S2.** Three‐week moving average of number of admissions with severe acute respiratory infection (SARI) by diagnostic code group, Norway, 28 September 2020–31 March 2024.
**Figure S3.** Weekly number of admissions with severe acute respiratory infection (SARI) where the patient was tested for SARS‐CoV‐2, influenza virus or respiratory syncytial virus (RSV), by definition for ‘tested’, Norway, 28 September 2020–31 March 2024.
**Figure S4.** Weekly proportion of admissions with severe acute respiratory infection (SARI) where the patient tested positive for SARS‐CoV‐2, influenza virus or respiratory syncytial virus (RSV), by definition for ‘tested’, Norway, 28 September 2020–31 March 2024.
**Figure S5.** Number of intensive care unit (ICU) admissions with severe acute respiratory infection (SARI) and positive PCR for SARS‐CoV‐2 (SARI‐COVID) and influenza virus (SARI‐influenza) in the surveillance dataset (NPR) by case definition: (1) intensive care (procedure code B0050), (2) invasive or non‐invasive ventilatory support (procedure codes GXAV01, GXAV10 and GXAV20) and (3) intensive care and/or invasive or non‐invasive ventilatory support, compared to ICU admissions with confirmed influenza registered in the Norwegian Intensive Care Registry (NIR), Norway, 3 January 2022–31 March 2024.
**Figure S6.** Weekly number of intensive care unit (ICU) admissions with severe acute respiratory infection (SARI) by case definition: (1) intensive care (procedure code B0050), (2) invasive or non‐invasive ventilatory support (procedure codes GXAV01, GXAV10 and GXAV20) and (3) intensive care and/or invasive or non‐invasive ventilatory support, Norway, 3 January 2022–31 March 2024.
**Figure S7.** Weekly number of intensive care unit (ICU) admissions with severe acute respiratory infection (SARI) where the patient received invasive or non‐invasive ventilatory support (procedure codes GXAV01, GXAV10 and GXAV20) without other types of intensive care; intensive care (procedure code B0050) without ventilatory support; and intensive care and invasive or non‐invasive ventilatory support, Norway, 3 January 2022–31 March 2024.
**Figure S8.** Weekly number of intensive care unit (ICU) admissions with severe acute respiratory infection (SARI) and positive PCR for SARS‐CoV‐2 (SARI‐COVID) in the surveillance dataset (NPR) by case definition: (1) intensive care (procedure code B0050), (2) invasive or non‐invasive ventilatory support (procedure codes GXAV01, GXAV10 and GXAV20) and (3) intensive care and/or invasive or non‐invasive ventilatory support, compared to ICU admissions with confirmed COVID‐19 registered in the Norwegian Intensive Care Registry (NIR), Norway, 3 January 2022–31 March 2024.
**Figure S9.** Weekly number of intensive care unit (ICU) admissions with severe acute respiratory infection (SARI) and positive PCR for influenza virus (SARI‐ influenza) in the surveillance dataset (NPR) by case definition: (1) intensive care (procedure code B0050), (2) invasive or non‐invasive ventilatory support (procedure codes GXAV01, GXAV10 and GXAV20) and (3) intensive care and/or invasive or non‐invasive ventilatory support, compared to ICU admissions with confirmed influenza registered in the Norwegian Intensive Care Registry (NIR), Norway, 3 January 2022–31 March 2024.
**Figure S10.** Weekly number of deaths associated with admission with severe acute respiratory infection (SARI) by case definition for death: (1) in‐hospital and (2) in‐hospital or ≤ 14 days after discharge, Norway, 28 September 2020–31 March 2024.
**Figure S11.** Nowcasted weekly admissions with severe acute respiratory infection (SARI), SARI‐COVID‐19, SARI‐influenza and SARI‐RSV with 50% and 95% prediction intervals, Norway, 1 December 2023–1 April 2024. The green line shows the number of reported admissions at the time the nowcasting was performed, and the orange line shows the final number of admissions after 5 weeks.
**Box S1.** Key demographics of Norway as of 1 January 2024. *Source:* Statistics Norway.

## Data Availability

The datasets analysed in the study contain individual‐level linked data from national registries in Norway. The public health experts had access to the data through Beredt C19, housed at the Norwegian Institute of Public Health. In Beredt C19, only fully anonymised data (i.e., data that are neither directly nor potentially indirectly identifiable) are permitted to be shared publicly. Legal restrictions therefore hinder public sharing of the dataset used in the study that would enable others to replicate the study findings. However, external researchers are freely able to request access to linked data from the same registries from outside the structure of Beredt C19, as per the normal procedure for conducting health research on registry data in Norway. Further information on Beredt C19, including contact information for the Beredt C19 project manager and information on access to data from each individual data source, is available at https://www.fhi.no/en/id/infectious‐diseases/coronavirus/emergency‐preparedness‐register‐for‐covid‐19/ and https://helsedata.no/en/.
