# Supplementary material for: Registry‐Based Surveillance of Severe Acute Respiratory Infections in Norway During 2021–2024
Source: Influenza Other Respir Viruses. 2025 Feb 14;19(2):e70080. doi: 10.1111/irv.70080 (PMC11826439; doi:10.1111/irv.70080)
Supplement: Supplementary file 1 — Table S1 International Classification of Diseases (ICD)‐10 codes in the Norwegian patient registry (NPR) included in the routine surveillance of severe acute respiratory infections (SARI), Norway, 2021–2023. Table S2. Length of stay for admissions with severe acute respiratory infection (SARI) by age group, Norway, 28 September 2020–31 March 2024. LQ = lower quartile, UQ = upper quartile. Table S3. Number and proportion of admissions linking to hospital admissions by type of admission (all‐cause and SARI), pathogen, test result and timing of test, Norway, 28 September 2020–31 March 2024. Table S4. Number and proportion of admissions with severe acute respiratory infection (SARI) where the patient was tested and tested positive for SARS‐CoV‐2, influenza virus and respiratory syncytial virus (RSV), by definition for ‘tested’, Norway, 28 September 2020–31 March 2024 (total number of admissions with SARI among patients with a permanent Norwegian ID = 212,933). Table S5. Sensitivity, specificity and positive and negative predictive value (PPV, NPV) of different case definitions for intensive care admissions with severe acute respiratory infection (SARI) and positive PCR for SARS‐CoV‐2 (SARI‐COVID) or for influenza virus (SARI‐influenza), compared to ICU admissions with confirmed COVID‐19 or influenza registered in the Norwegian Intensive Care Registry (NIR, NCOVID‐19 = 1530, Ninfluenza = 416), Norway, 3 January 2022–31 March 2024. Table S6. Number and proportion of deaths associated with admission with severe acute respiratory infection (SARI) where a diagnostic code for SARI was registered in the death certificate, by timing of death in relation to the hospital stay, Norway, 28 September 2020–31 March 2024. Table S7. Number and proportion of admissions with severe acute respiratory infection (SARI) where the patient was tested for SARS‐CoV‐2, influenza virus and respiratory syncytial virus (RSV) by age group, Norway, 28 September 2020–31 March 2024. Only SARI cases with [file IRV-19-e70080-s001.docx]

**Supplementary material for the article Registry-based surveillance of severe acute respiratory infections in Norway during 2021-2024**

Table of contents

[1. The Norwegian population and health care system 1](#_Toc188859131)

[2. The key data sources in the Norwegian SARI surveillance system 2](#_Toc188859132)

[2.1 The Norwegian Patient Registry 2](#_Toc188859133)

[2.2 Norwegian Surveillance System for Communicable Diseases (MSIS) laboratory database 3](#_Toc188859134)

[2.3 The National Population Registry 3](#_Toc188859135)

[3. International Classification of Diseases (ICD)-10 codes included in the case definition for SARI 3](#_Toc188859136)

[4. Evaluation of selected attributes 5](#_Toc188859137)

[4.1 Case definitions 5](#_Toc188859138)

[4.2 Testing strategy and representativeness of testing 19](#_Toc188859139)

[5. Nowcasting 19](#_Toc188859140)

[6. References 21](#_Toc188859141)

## **The Norwegian population and health care system**

Norway has a population of 5.6 million (1). Key demographics of Norway, including the population density and structure are described in Box S1.

**Box S1. Key demographics of Norway as of 1 January 2024. Source: Statistics Norway.**

| 0-19 years: 1,244,282  20-64 years: 3,269,753  ≥65 years: 1,036,168  Total population (population density): 5,550,203 (17/km^2^)  Percentage of the population living in the capital (population density): 20% (3,974/km2)  Percentage of the population living in the 4 largest cities excluding the capital (population density): 15% (2,942/km2)  Percentage of males: 50.4%  Percentage of females: 49.6%  Percentage of developed land: 1.7%  Percentage of agricultural land: 3.5% |
| --- |

Norway has universal health coverage, funded primarily by general taxes and by payroll contributions shared by employers and employees. Services covered include primary, ambulatory, mental health, and hospital care, as well as selected outpatient prescription drugs. Patients make copayments for some services and products, with caps on out-of-pocket contributions for most services. Municipalities organize primary health care, while the national government is responsible for specialized care, including hospital services, through the state-owned regional health authorities (2). Most hospitals in Norway are public hospitals, funded and owned by the state. All Norwegian citizens can choose their general practitioner (GP) from a list. Outpatient doctors act as gatekeepers for specialized care (3). To enter a hospital emergency room, referrals from primary care physicians are most often needed, unless the patient has contacted the emergency telephone number and comes directly by ambulance.

In Norway, there are some special arrangements for treating the elderly, which may lead to this patient group being somewhat underrepresented in the SARI surveillance. Firstly, nursing home residents often receive treatment for respiratory infections in the institution in which they live. Secondly, there are certain local arrangements where elderly living at home may receive short time care in local nursing homes for treatments of clarified conditions, this to avoid hospitalisations. Thirdly, some municipalities have a “municipality hospital” which accepts patients for shorter admissions when the diagnosis is clear. Since these arrangements are organized as a part of primary health care, they are not captured by the SARI surveillance system which is based on admissions in specialized health care.

## **The key data sources in the Norwegian SARI surveillance system**

### **The Norwegian Patient Registry**

The Norwegian Patient Registry (NPR) was established in 2008 and was previously owned by the Norwegian Directorate of Health, but since 1.1.2024 the registry has been owned by the Norwegian Institute of Public Health (NIPH). NPR is a mandatory national registry with data on all patients who are waiting for or have received health care in the publicly funded specialist health services in Norway. NPR contains data on administration, somatic and psychiatric care, rehabilitation, and injuries. The data are individual-level, with a unique person-ID that allows for linkage with other data sources. Data is collected from electronic patient records and normally transferred to NPR on a monthly basis, however, during the COVID-19 pandemic, data has been transferred daily. The main aim of the registry is to form the basis for administration, management, and quality assurance of specialist healthcare services, including funding. Therefore, there are certain limitations related to using the data for surveillance purposes.

NPR is the key data source to identifying hospital admissions with SARI. The registry contains data on hospital admissions with personal, administrative, and medical information. Relevant variables include admission and discharge date, urgency, medical procedures, including ventilatory support and intensive care, ICD-10 diagnoses at discharge and status at discharge (alive/dead).

Together with the data from the Norwegian Registry for Primary Health Care (NRPC), the data from NPR can also be used for defining medical risk groups for severe acute respiratory infections.

Even though privately funded hospitals do not report to NPR, it can be assumed that NPR captures nearly all admissions in specialist health care with SARI, as most of the privately funded hospitals provide elective services such as surgery and rehabilitation.

### **Norwegian Surveillance System for Communicable Diseases (MSIS) laboratory database**

The MSIS laboratory database was established in April of 2020 and is owned by the NIPH. Reporting of PCR test results for SARS-CoV-2 commenced immediately, and since September 2020, all Norwegian microbiological laboratories have been required to report PCR test results for pathogens defined as COVID-19 related in the regulation on the Norwegian Surveillance System for Communicable Diseases, namely adenovirus, human metapneumovirus, influenza virus, parainfluenza virus, rhinovirus, respiratory syncytial virus (RSV), *Bordetella pertussis, Chlamydia pneumoniae* and *Mycoplasma pneumoniae*. Since September 2021, all PCR test results are to be reported to this mandatory nationwide registry. As of April 2024, 25 out of 26 laboratories send all microbiological results (including negative and positive) to the MSIS laboratory database. The data are individual-level with a unique person-ID that allows for linkage with other data sources for some pathogens, however there are some limitations on storage of personal identifiable information on all microbiology results. Directly identifiable characteristics for positive or negative COVID-19 related test results must not be deleted, but this is a temporary legal authorization. The registry is fully electronic, and data are reported on an ongoing basis. The main aim of the registry is to monitor infectious diseases in humans in Norway.

The data relevant to the SARI surveillance system include demographic data, date of testing and reporting, laboratory, pathogen, type of sample, requisitioning person/ unit, and result. The data allow the identification of individuals who tested positive or negative for different respiratory pathogens and thus identify individuals hospitalised with laboratory-confirmed infection.

### **The National Population Registry**

The National Population Registry is maintained by the Norwegian Tax Administration and contains information of everyone that resides or have resided in Norway. The registry forms the basis for the tax registry, the electoral registry and population statistics. The registry was established in 1964. The data are individual-level with a unique person-ID that allows for linkage with other data sources. The aim of the registry is to provide information for official tasks and public administration, research, statistics and to look after basic societal needs.

The data relevant for the SARI surveillance system in this registry include e.g., dates of birth and death, sex, place of birth and residence, citizenship, resident status, civil status, and family relations.

## **International Classification of Diseases (ICD)-10 codes included in the case definition for SARI**

**Table S1. International Classification of Diseases (ICD)-10 codes in the Norwegian patient registry (NPR) included in the routine surveillance of severe acute respiratory infections (SARI), Norway, 2021-2023.**

| **Disease group** | **ICD-10 code** | **Description** |
| --- | --- | --- |
| **Certain infectious and parasitic diseases - A** | | |
| Whooping cough | A37.0 | Whooping cough due to Bordetella pertussis |
|  | A37.1 | Whooping cough due to Bordetella parapertussis |
|  | A37.8 | Whooping cough due to other Bordetella species |
|  | A37.9 | Whooping cough, unspecified species |
| **Diseases of the eye and adnexa, Diseases of the ear and mastoid process - H** | | |
| Otitis media | H65.0 | Acute serous otitis media |
|  | H65.1 | Other acute nonsuppurative otitis media |
|  | H65.9 | Unspecified nonsuppurative otitis media |
|  | H66.0 | Acute suppurative otitis media |
|  | H66.4 | Suppurative otitis media, unspecified |
|  | H66.9 | Otitis media, unspecified |
|  | H67.0 | Otitis media in bacterial diseases classified elsewhere |
|  | H67.1 | Otitis media in viral diseases classified elsewhere |
|  | H67.8 | Otitis media in other diseases classified elsewhere |
| **Diseases of the respiratory system - J** | | |
| Acute upper respiratory infections | J00 | Acute nasopharyngitis [common cold] |
|  | J01.0 | Acute maxillary sinusitis |
|  | J01.1 | Acute frontal sinusitis |
|  | J01.2 | Acute ethmoidal sinusitis |
|  | J01.3 | Acute sphenoidal sinusitis |
|  | J01.4 | Acute pansinusitis |
|  | J01.8 | Other acute sinusitis |
|  | J01.9 | Acute sinusitis, unspecified |
|  | J02.0 | Streptococcal pharyngitis |
|  | J02.8 | Acute pharyngitis due to other specified organisms |
|  | J02.9 | Acute pharyngitis, unspecified |
|  | J03.0 | Streptococcal tonsillitis |
|  | J03.8 | Acute tonsillitis due to other specified organisms |
|  | J03.9 | Acute tonsillitis, unspecified |
|  | J04.0 | Acute laryngitis |
|  | J04.1 | Acute tracheitis |
|  | J04.2 | Acute laryngotracheitis |
|  | J05.0 | Acute obstructive laryngitis [croup] |
|  | J05.1 | Acute epiglottitis |
|  | J06.0 | Acute laryngopharyngitis |
|  | J06.8 | Other acute upper respiratory infections of multiple sites |
|  | J06.9 | Acute upper respiratory infection, unspecified |
| Acute lower respiratory infections, including influenza | J09 | Influenza due to identified zoonotic or pandemic influenza viruses |
|  | J10.0 | Influenza due to identified seasonal influenza virus with pneumonia |
|  | J10.1 | Influenza due to identified seasonal influenza virus with other respiratory manifestations |
|  | J10.8 | Influenza due to identified seasonal influenza virus with other manifestations |
|  | J11.0 | Influenza due to unidentified influenza virus with pneumonia |
|  | J11.1 | Influenza due to unidentified influenza virus with other respiratory manifestations |
|  | J11.8 | Influenza due to unidentified influenza virus with other manifestations |
|  | J12.0 | Adenoviral pneumonia |
|  | J12.1 | Respiratory syncytial virus pneumonia |
|  | J12.2 | Parainfluenza virus pneumonia |
|  | J12.3 | Human metapneumovirus pneumonia |
|  | J12.8 | Other viral pneumonia |
|  | J12.9 | Viral pneumonia, unspecified |
|  | J13 | Pneumonia due to Streptococcus pneumoniae |
|  | J14 | Pneumonia due to Hemophilus influenzae |
|  | J15.0 | Pneumonia due to Klebsiella pneumoniae |
|  | J15.1 | Pneumonia due to Pseudomonas |
|  | J15.2 | Pneumonia due to staphylococcus |
|  | J15.3 | Pneumonia due to streptococcus, group B |
|  | J15.4 | Pneumonia due to other streptococci |
|  | J15.5 | Pneumonia due to Escherichia coli |
|  | J15.6 | Pneumonia due to other Gram-negative bacteria |
|  | J15.7 | Pneumonia due to Mycoplasma pneumoniae |
|  | J15.8 | Pneumonia due to other specified bacteria |
|  | J15.9 | Unspecified bacterial pneumonia |
|  | J16.0 | Chlamydial pneumonia |
|  | J16.8 | Pneumonia due to other specified infectious organisms |
|  | J17.0 | Pneumonia in bacterial diseases classified elsewhere |
|  | J17.1 | Pneumonia in viral diseases classified elsewhere |
|  | J17.2 | Pneumonia in fungal diseases |
|  | J17.3 | Pneumonia in parasitic diseases |
|  | J17.8 | Pneumonia in other diseases classified elsewhere |
|  | J18.0 | Bronchopneumonia, unspecified organism |
|  | J18.1 | Lobar pneumonia, unspecified organism |
|  | J18.2 | Hypostatic pneumonia, unspecified organism |
|  | J18.8 | Other pneumonia, unspecified organism |
|  | J18.9 | Pneumonia, unspecified organism |
|  | J20.0 | Acute bronchitis due to Mycoplasma pneumoniae |
|  | J20.1 | Acute bronchitis due to Hemophilus influenzae |
|  | J20.2 | Acute bronchitis due to streptococcus |
|  | J20.3 | Acute bronchitis due to coxsackievirus |
|  | J20.4 | Acute bronchitis due to parainfluenza virus |
|  | J20.5 | Acute bronchitis due to respiratory syncytial virus |
|  | J20.6 | Acute bronchitis due to rhinovirus |
|  | J20.7 | Acute bronchitis due to echovirus |
|  | J20.8 | Acute bronchitis due to other specified organisms |
|  | J20.9 | Acute bronchitis, unspecified |
|  | J21.0 | Acute bronchiolitis due to respiratory syncytial virus |
|  | J21.1 | Acute bronchiolitis due to human metapneumovirus |
|  | J21.8 | Acute bronchiolitis due to other specified organisms |
|  | J21.9 | Acute bronchiolitis, unspecified |
|  | J22 | Unspecified acute lower respiratory infection |
| Acute respiratory distress syndrome in adults [ARDS] | J80 |  |
| **Codes for special purposes - U** | | |
| COVID-19 | U07.1 | COVID-19 with identified virus |
|  | U07.2 | COVID-19 without identified virus |

## **Evaluation of selected attributes**

### **Case definitions**

#### Definition of SARI based on ICD-10 codes

Figure S1 compares the routinely used case definition including acute upper and lower respiratory infections (URI, LRI), COVID-19, acute respiratory distress syndrome in adults (ARDS), pertussis, and acute and unspecified otitis media with two alternative versions: URI, LRI and COVID-19; and LRI and COVID-19 over time.


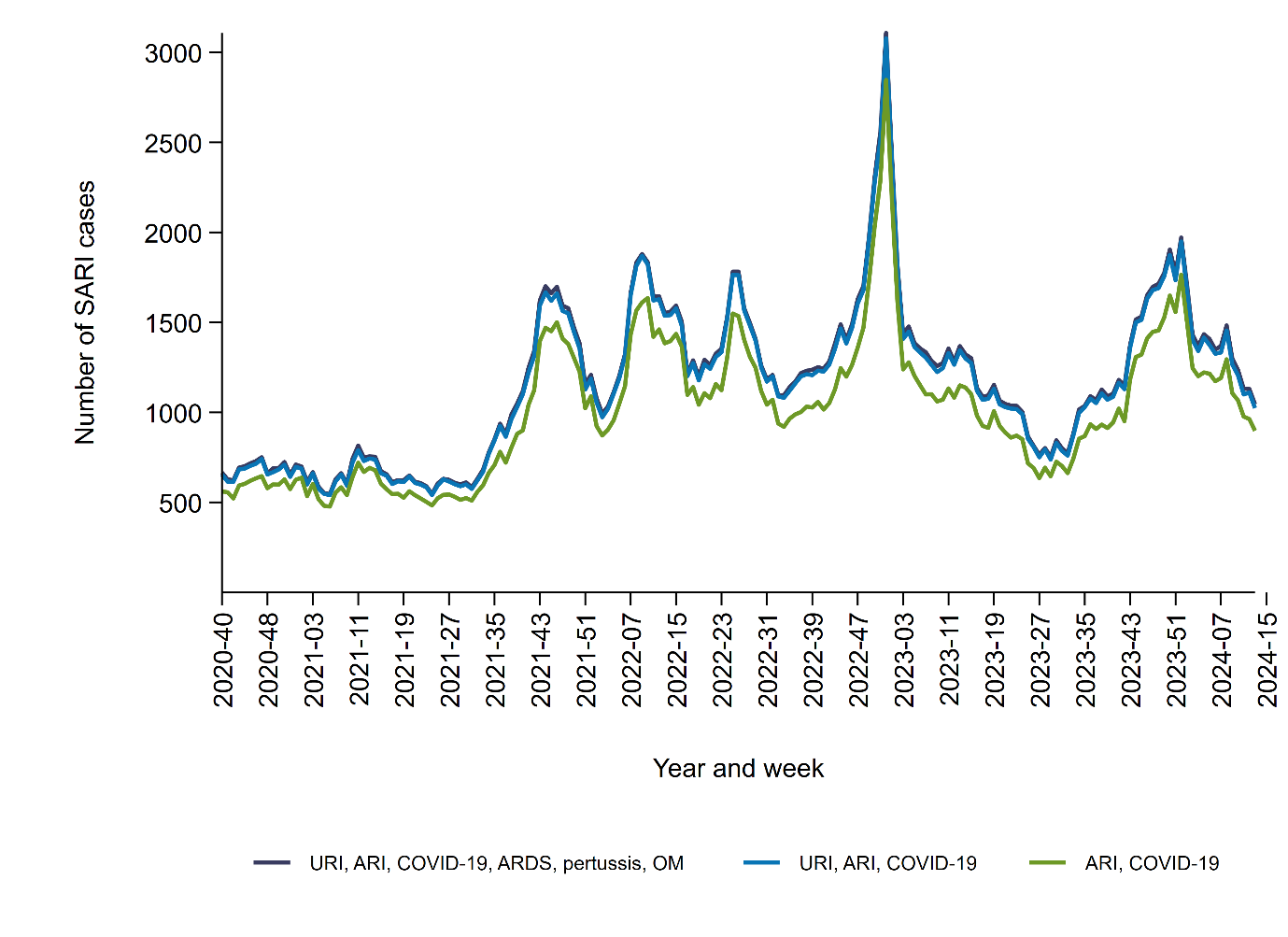
**Figure S1. Weekly number of admissions with severe acute respiratory infection (SARI) by case definition, Norway, 28.9.2020 – 31.3.2024.**

URI: upper respiratory infection (J00-J06); LRI: lower respiratory infection (J09-J22); COVID-19: U07.1, U07.2; ARDS: acute respiratory distress syndrome in adults (J80); Pertussis: A37; OM: acute and unspecified otitis media (H65-H67)

We also plotted the 3-week moving average of number of hospital admissions by diagnostic code category (J00, J01, J02 etc.) to assess how these affected the burden of SARI.

The most frequently registered diagnostic codes included those for confirmed COVID-19 (U07.1), bacterial pneumonia (J15), confirmed influenza (J10), bronchiolitis (J21), pneumonia with unspecified organism (J18), acute bronchitis (J20) and acute URI of multiple and unspecified sites (J06) (Figure S2), and most of the peaks seen for these codes coincided with the peaks detected for SARI-COVID-19, SARI-influenza and/or SARI-RSV. Some codes in the LRI group were registered only infrequently; the numbers of admissions with Chlamydial pneumonia (J16) and pneumonia in diseases classified elsewhere (J17) remained low and stable throughout the study period. Despite of this, they should still be included in the case definition due to their corresponding pathogens’ propensities to cause outbreaks.


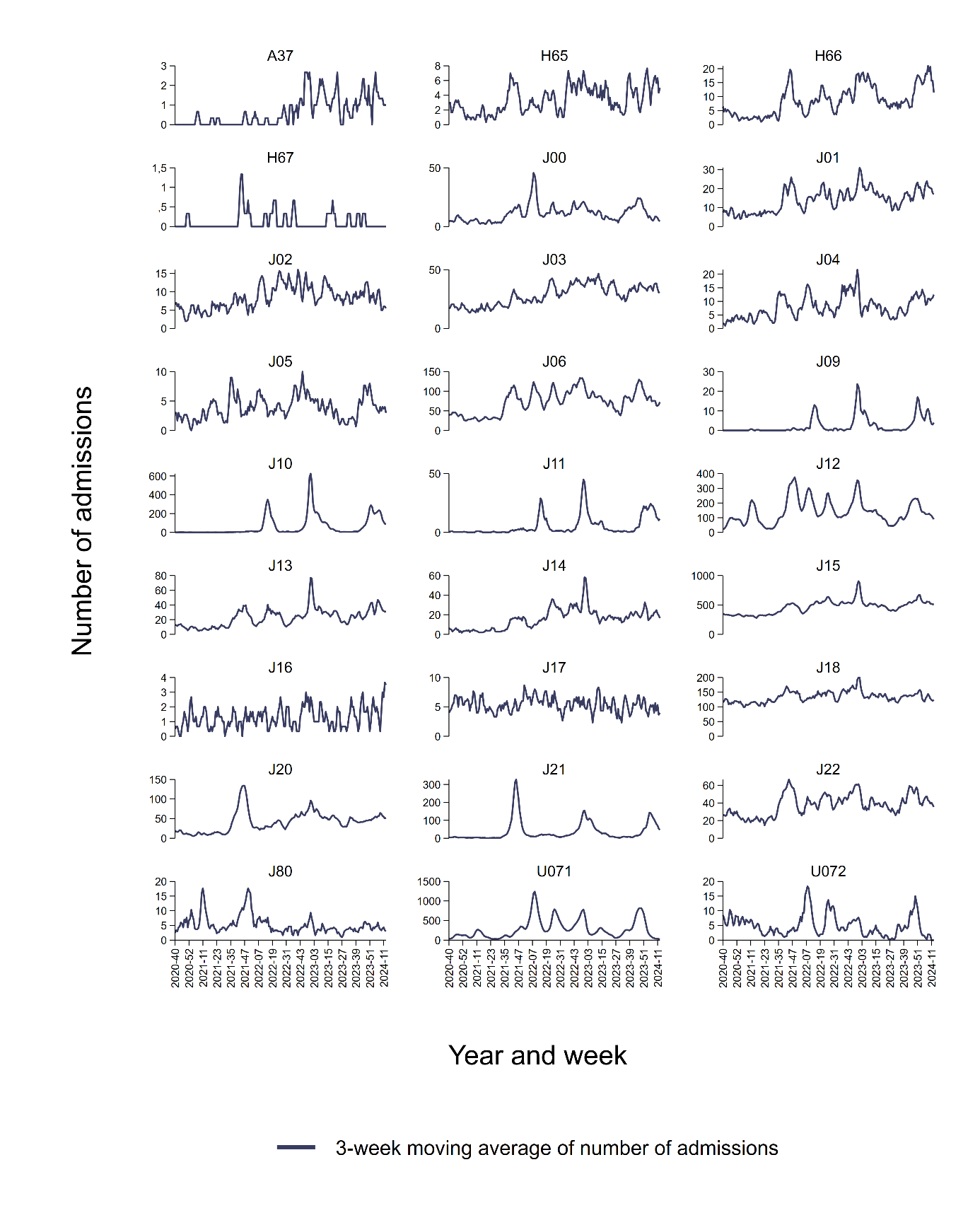


**Figure S2. 3-week moving average of number of admissions with severe acute respiratory infection (SARI) by diagnostic code group, Norway, 28.9.2020 – 31.3.2024.**

*A37 Pertussis; H65 Non-suppurative otitis media; H66 Suppurative and unspecified otitis media; H67 Otitis media in diseases classified elsewhere; J00 Acute nasopharyngitis (common cold); J01 Acute sinusitis; J02 Acute pharyngitis; J03 Acute tonsillitis; J04 Acute laryngitis and tracheitis; J05 Acute obstructive laryngitis (croup) and epiglottitis; J06 Acute upper respiratory infections of multiple and unspecified sites; J09 Influenza due to identified zoonotic or pandemic influenza virus; J10 Influenza due to identified seasonal influenza virus; J11 Influenza, virus not identified; J12 Viral pneumonia, not elsewhere classified; J13 Pneumonia due to *Streptococcus pneumoniae*; J14 Pneumonia due to *Haemophilus influenzae*; J15 Bacterial pneumonia, not elsewhere classified; J16 Pneumonia due to other infectious organisms, not elsewhere classified; J17 Pneumonia in diseases classified elsewhere; J18 Pneumonia, unspecified organism; J20 Acute bronchitis; J21 Acute bronchiolitis; J22 Unspecified acute lower respiratory infection; J80 Acute respiratory distress syndrome; U07.1 COVID-19, virus identified; U07.2 COVID-19, virus not identified

#### Definition of hospitalisation

There are several ways to identify/ define hospitalised patients in registry data. While some countries have used a length of stay of >12 or >24 hours for this (4-6), the Norwegian SARI surveillance system relied on a variable that categorizes different types of contacts in specialist health care: admissions, day treatment, and policlinical contacts (7). Contacts registered as admissions were included in the SARI surveillance, regardless of length of stay. Other types of contacts, even those with a duration of >12 or >24 hours, were not included. To assess the performance of this method of identifying hospitalised patients, we enriched the surveillance dataset by including also policlinical contacts and day treatment with a SARI diagnosis registered. We excluded contacts with missing discharge date and calculated the median (lower – upper quartile (LQ – UQ)) length of stay by age group for admissions with SARI. We also counted the number of policlinical contacts and day treatment registered with a SARI diagnosis where the duration of the contact was >12 and >24 hours. To assess if the system missed deaths with SARI that occur in e.g., the emergency room, we counted the number of deaths with SARI for policlinical contacts and day treatment.

The median (LQ – UQ) length of stay among the SARI cases was 93 (46-176) hours. The age groups 0-4 and 5-14 years had the shortest length of stay (Table S2), and 11% and 12% of the admissions had a length of stay <12 hours. The longest median length of stay was recorded for the elderly (Table S2). Of the other types of contacts where a SARI diagnosis was registered, 516 (0.2% of the total number of admissions with SARI [214775]), had a duration of >12 hours, and of these, 129 were >24 hours. Almost all deaths with SARI were recorded among patients registered as admitted; only 6 deaths were among patients registered as not admitted.

**Table S2. Length of stay for admissions with severe acute respiratory infection (SARI) by age group, Norway, 28.9.2020 – 31.3.2024. LQ: lower quartile, UQ: upper quartile**

|  |  | **Length of stay in hours** | | |
| --- | --- | --- | --- | --- |
| **Age group** | **n** | **median** | **LQ** | **UQ** |
| 0-4 years | 23,685 | 31 | 18 | 71 |
| 5-14 years | 4,983 | 36 | 18 | 73 |
| 15-29 years | 10,737 | 48 | 24 | 96 |
| 30-64 years | 46,877 | 86 | 44 | 174 |
| 65-79 years | 68,912 | 116 | 67 | 218 |
| 80+ years | 59,581 | 115 | 67 | 194 |
| Total | 214,775 | 93 | 46 | 176 |

To summarize, by including all SARI cases that are categorized as “admitted”, we were able to capture also children who sometimes had very short stays <12 hours. We did miss a very small number of other types of contacts that had a duration of >12 or even >24 hours, but importantly, the number of deaths missed was <10. As long as hospitals follow the guidelines for the categorization of patient contacts (7), using this variable for defining hospital admissions in Norway is justified.

#### Definition of “tested” and “laboratory-confirmed”

For linking data on hospitalisation with laboratory test results, samples taken ≤14 days before admission and ≥2 days after discharge are routinely included in the surveillance. As diagnostic codes are registered usually at discharge, it is not possible to reliably distinguish between admissions due to SARI and hospital acquired infections. Furthermore, the surveillance system aims to capture the full burden of SARI on health care, as cases often require isolation, regardless of place of infection. Therefore, it was considered important to include tests taken during the hospital stay, in addition to those taken before admission. For the definition of a SARI case tested for a SARS-CoV-2, influenza virus and RSV, we assessed two additional time windows for linking laboratory tests to hospitals stays and compared them to the one currently used: ≤14 days before admission and up to discharge, and ≤14 days before and ≥2 days after admission. Even though antigen rapid test results for SARS-CoV-2 are included in routine surveillance, they were excluded from these assessments due to the very low number of SARI cases tested only with a rapid antigen test (n=259). We also excluded cases without a permanent Norwegian ID to ensure that data on hospitalisation could be linked correctly to data on testing.

More than 93%, 93% and 91% of the samples for SARS-CoV-2, influenza virus and RSV, respectively, included in the surveillance dataset were taken within the time period of ≤14 days before and ≤2 days after admission. These percentages were even higher for SARI cases, and for positive tests. Depending on the pathogen, 6-8% of all the samples were taken later during the admission, and ≤1% were taken right after discharge (table S3). The percentage of SARI cases tested and positive for each of the three pathogens was similar for all three definitions (table S4, figures S1 and S2).

**Table S3. Number and proportion of admissions linking to hospital admissions by type of admission (all-cause and SARI), pathogen, test result and timing of test, Norway, 28.9.2020 – 31.3.2024**

| **Pathogen** | **Test result** | **Timing of test** | **All-cause admissions** | | **SARI cases** | |
| --- | --- | --- | --- | --- | --- | --- |
|  |  |  | **n** | **%** | **n** | **%** |
| **SARS-CoV-2** | | |  |  |  |  |
|  | Positive or negative | ≤14 days before and ≤2 days after admission | 633,886 | 93.2 | 167,518 | 94.9 |
|  |  | >2 days after admission and up to discharge date | 39,761 | 5.8 | 8,714 | 4.9 |
|  |  | 1-2 days after discharge | 6,704 | 1.0 | 263 | 0.1 |
|  | Positive | ≤14 days before and ≤2 days after admission | 59,624 | 92.9 | 46,713 | 94.1 |
|  |  | >2 days after admission and up to discharge date | 4,135 | 6.4 | 2,845 | 5.7 |
|  |  | 1-2 days after discharge | 437 | 0.7 | 58 | 0.1 |
| **Influenza virus** | | |  |  |  |  |
|  | Positive or negative | ≤14 days before and ≤2 days after admission | 440,812 | 93.1 | 148,167 | 94.4 |
|  |  | >2 days after admission and up to discharge date | 29,462 | 6.2 | 8,538 | 5.4 |
|  |  | 1-2 days after discharge | 3,096 | 0.7 | 209 | 0.1 |
|  | Positive | ≤14 days before and ≤2 days after admission | 14,302 | 96.3 | 12,554 | 97.3 |
|  |  | >2 days after admission and up to discharge date | 502 | 3.4 | 343 | 2.7 |
|  |  | 1-2 days after discharge | 51 | 0.3 | 6 | 0.0 |
| **Respiratory syncytial virus (RSV)** | | |  |  |  |  |
|  | Positive or negative | ≤14 days before and ≤2 days after admission | 287,365 | 91.0 | 104,288 | 92.3 |
|  |  | >2 days after admission and up to discharge date | 26,212 | 8.3 | 8,517 | 7.5 |
|  |  | 1-2 days after discharge | 2,276 | 0.7 | 239 | 0.2 |
|  | Positive | ≤14 days before and ≤2 days after admission | 11,891 | 96.9 | 10,085 | 97.9 |
|  |  | >2 days after admission and up to discharge date | 339 | 2.8 | 209 | 2.0 |
|  |  | 1-2 days after discharge | 38 | 0.3 | 6 | 0.1 |

**Table S4. Number and proportion of admissions with severe acute respiratory infection (SARI) where the patient was tested and tested positive for SARS-CoV-2, influenza virus and respiratory syncytial virus (RSV), by definition for “tested”, Norway, 28.9.2020 – 31.3.2024 (total number of admissions with SARI among patients with a permanent Norwegian ID = 212,933)**

| **Pathogen** | **Definition** | **SARI cases tested** | | **SARI cases positive** | |
| --- | --- | --- | --- | --- | --- |
|  |  | **n** | **%** | **n** | **%** |
| **SARS-CoV-2** | |  |  |  |  |
|  | ≤14 days before admission & ≤2 days after discharge | 167,518 | 78.0 | 46,713 | 27.9 |
|  | ≤14 days before admission & during hospital stay | 176,126 | 82.0 | 49,526 | 28.1 |
|  | ≤14 days before & ≤2 days after admission | 176,495 | 82.2 | 49,616 | 28.1 |
| **Influenza virus** | |  |  |  |  |
|  | ≤14 days before admission & ≤2 days after discharge | 148,167 | 69.0 | 12,554 | 8.5 |
|  | ≤14 days before admission & during hospital stay | 156,616 | 72.9 | 12,889 | 8.2 |
|  | ≤14 days before & ≤2 days after admission | 156,914 | 73.1 | 12,903 | 8.2 |
| **RSV** | |  |  |  |  |
|  | ≤14 days before admission & ≤2 days after discharge | 104,288 | 48.6 | 10,085 | 9.7 |
|  | ≤14 days before admission & during hospital stay | 112,723 | 52.5 | 10,279 | 9.1 |
|  | ≤14 days before & ≤2 days after admission | 113,044 | 52.6 | 10,300 | 9.1 |


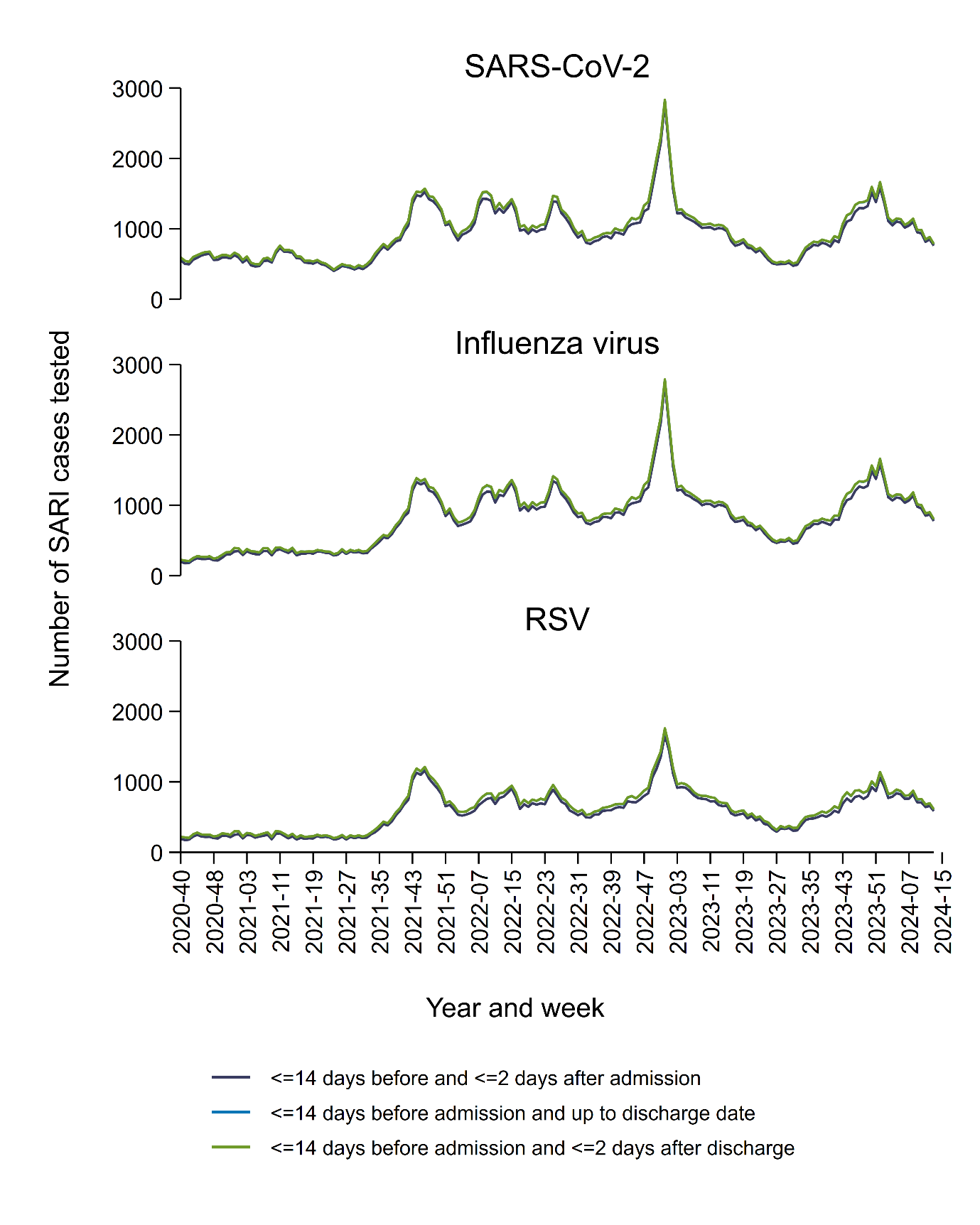


**Figure S3. Weekly number of admissions with severe acute respiratory infection (SARI) where the patient was tested for SARS-CoV-2, influenza virus or respiratory syncytial virus (RSV), by definition for “tested”, Norway, 28.9.2020 – 31.3.2024**


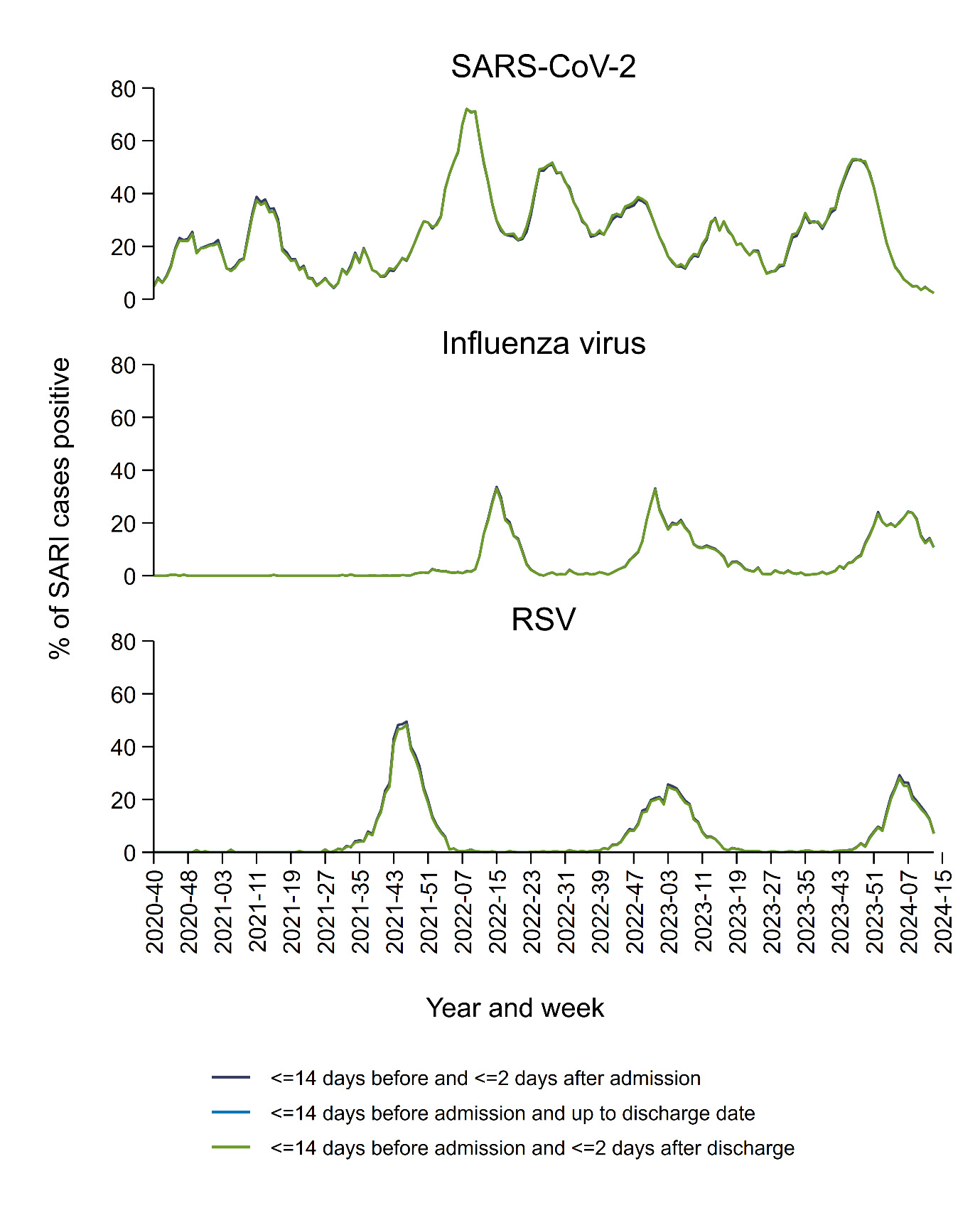


**Figure S4. Weekly proportion of admissions with severe acute respiratory infection (SARI) where the patient tested positive for SARS-CoV-2, influenza virus or respiratory syncytial virus (RSV), by definition for “tested”, Norway, 28.9.2020 – 31.3.2024**

To summarize, the percentages of SARI cases positive for SARS-CoV-2, influenza virus and RSV were similar in the studied time windows, while tests taken 1-2 days after discharge contributed very little in terms of number of cases tested and positive. Including only tests taken ≤14 days before and ≤2 days after admission is common especially in studies aiming to primarily capture hospital admissions due to the pathogen of interest (8, 9). In this surveillance system based on discharge codes where primary codes cannot be trusted to reliably identify admissions due to SARI, it is inevitable that also hospital acquired infections are included among the SARI cases. Therefore, we consider it relevant to include also samples taken during the admission, while those taken shortly after discharge could be excluded.

#### Definition of ICU-SARI

Different kinds of medical procedures conducted in specialist health care are registered in NPR using the procedure coding system for medical procedures (NCMP). Before the COVID-19 pandemic, intensive care admissions were not registered in a systematic way in NPR. Up to the end of 2021, only procedure codes for invasive and noninvasive ventilatory support were available for use in the SARI surveillance, and they have served as a proxy for ICU admissions with SARI. In January 2022, a new procedure code for intensive care (IC) was introduced, with specific criteria for its use (7, 10). The criteria are the same as for the Norwegian Intensive Care Registry (NIR), which collects information on patients treated in Norwegian intensive care units (ICU) (10). In addition to the main data collection form that is filled out for all patients, separate forms are filled out for patients admitted to ICU with influenza and COVID-19. The data collection in NIR for patients admitted to ICU with influenza and COVID-19 is timely and thus the data can be used for surveillance purposes, while surveillance of other SARI patients through NIR is currently not possible.

Using the surveillance dataset, we described the number of SARI admissions with IC, ventilatory support, and IC and/or ventilatory support between 3.1.2022 and 31.3.2024. We assessed the accuracy of the proxy case definitions based on procedure codes for ventilatory support and IC for admissions with SARI-influenza and SARI-COVID-19. We considered ICU admissions registered in NIR with confirmed influenza and COVID-19 to be the gold standard. Admissions registered >90 days after the previous admission date in NIR were considered as new infection episodes. We merged these data to the surveillance dataset where only SARI admissions with positive test for influenza or SARS-CoV-2 were included. We considered the records in surveillance dataset and NIR match if the hospital stays registered in the two registries overlapped at least partially. We excluded cases without a permanent Norwegian ID, for whom data could not be linked reliably. We compared the weekly number of ICU admissions in the surveillance dataset and NIR over time, and calculated the sensitivity, specificity, positive predictive value (PPV) and negative predictive value (NPV) for the different proxy case definitions for ICU-SARI-influenza and ICU-SARI-COVID-19 (IC; ventilatory support; and IC and/or ventilatory support).

Between 3.1.2022 and 31.3.2024, a total of 16682 SARI cases received IC and/or ventilatory support. Of these, 7534 (45%) received both, 6410 (38%) received ventilatory support without IC and 2738 (16%) received IC without ventilatory support. The studied case definitions for ICU admissions with COVID-19 and influenza in the surveillance dataset and ICU admissions registered in NIR overlapped only partially. For both influenza and COVID-19, the studied case definitions had a specificity of ≥95% and NPV of 99%. The sensitivity was lowest and PPV highest for the case definition including only the procedure code for IC, while the combination of IC and/or ventilatory support yielded highest sensitivity and lowest PPV (table S5, figure S5). Figures S6-S9 show ICU admissions with SARI, SARI-COVID and SARI-influenza according to the different proxy case definitions over time, also compared with ICU admissions registered in NIR.

**Table S5. Sensitivity, specificity, positive and negative predictive value (PPV, NPV) of different case definitions for intensive care admissions with severe acute respiratory infection (SARI) and positive PCR for SARS-CoV-2 (SARI-COVID) or for influenza virus (SARI-influenza), compared to ICU admissions with confirmed COVID-19 or influenza registered in the Norwegian Intensive Care Registry (NIR, N_COVID-19_ = 1530, N_influenza_ = 416), Norway, 03.01.2022 – 31.3.2024.**

| **Case definition (procedure code(s))** | | **N** | **Sensitivity (%)** | **Specificity (%)** | **PPV (%)** | **NPV (%)** |
| --- | --- | --- | --- | --- | --- | --- |
| SARI-COVID | |  |  |  |  |  |
|  | B0050 | 2129 | 68.6 | 97.3 | 49.6 | 98.8 |
|  | GXAV01, GXAV10 and/or GXAV20 | 2679 | 65.9 | 95.8 | 37.8 | 98.6 |
|  | B0050, GXAV01, GXAV10 and/or GXAV20 | 3318 | 81.0 | 94.8 | 37.6 | 99.2 |
| SARI-influenza | |  |  |  |  |  |
|  | B0050 | 576 | 65.9 | 97.6 | 47.6 | 98.8 |
|  | GXAV01, GXAV10 and/or GXAV20 | 818 | 67.1 | 95.6 | 34.1 | 98.9 |
|  | B0050, GXAV01, GXAV10 and/or GXAV20 | 968 | 81.0 | 94.9 | 34.8 | 99.3 |


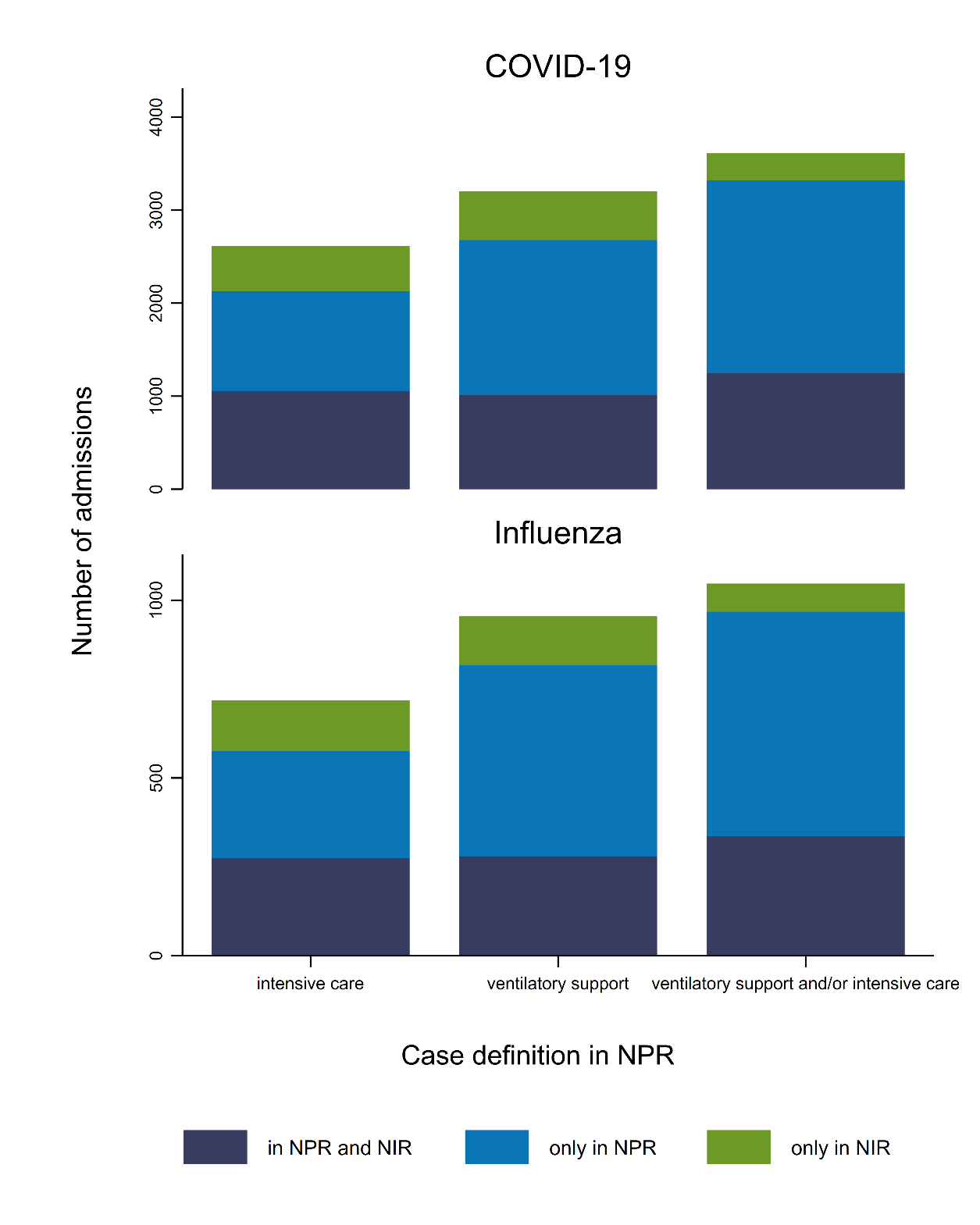


**Figure S5. Number of intensive care unit (ICU) admissions with severe acute respiratory infection (SARI) and positive PCR for SARS-CoV-2 (SARI- COVID) and influenza virus (SARI- influenza) in the surveillance dataset (NPR) by case definition: 1) intensive care (procedure code B0050), 2) invasive or non-invasive ventilatory support (procedure codes GXAV01, GXAV10, GXAV20) and 3) intensive care and/or invasive or non-invasive ventilatory support, compared to ICU admissions with confirmed influenza registered in the Norwegian Intensive Care Registry (NIR), Norway, 03.01.2022 – 31.3.2024**


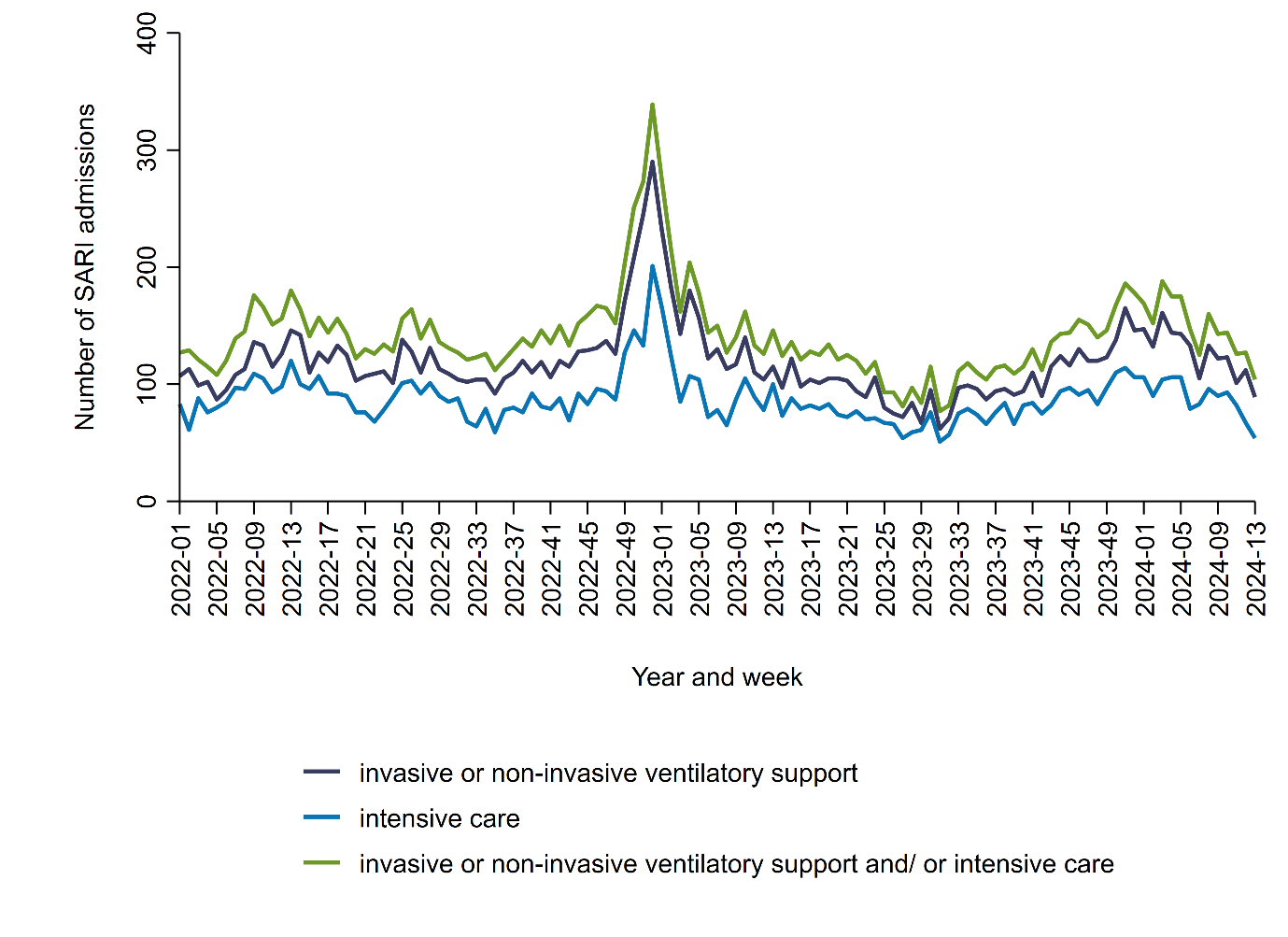


**Figure S6. Weekly number of intensive care unit (ICU) admissions with severe acute respiratory infection (SARI) by case definition: 1) intensive care (procedure code B0050), 2) invasive or non-invasive ventilatory support (procedure codes GXAV01, GXAV10, GXAV20) and 3) intensive care and/or invasive or non-invasive ventilatory support, Norway, 03.01.2022 – 31.3.2024**


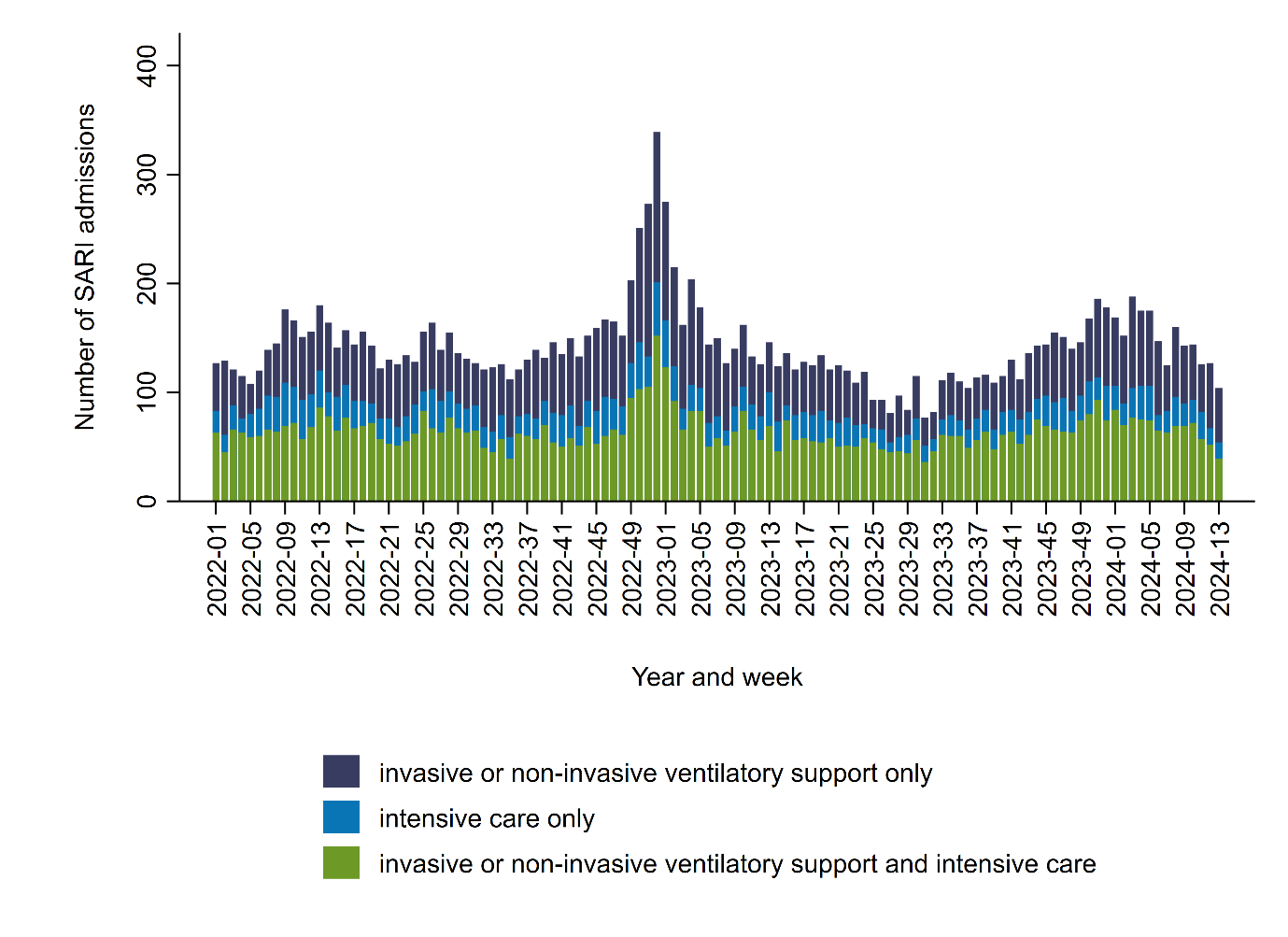


**Figure S7. Weekly number of intensive care unit (ICU) admissions with severe acute respiratory infection (SARI) where the patient received invasive or non-invasive ventilatory support (procedure codes GXAV01, GXAV10, GXAV20) without other types of intensive care; intensive care (procedure code B0050) without ventilatory support; and intensive care and invasive or non-invasive ventilatory support, Norway, 03.01.2022 – 31.3.2024**


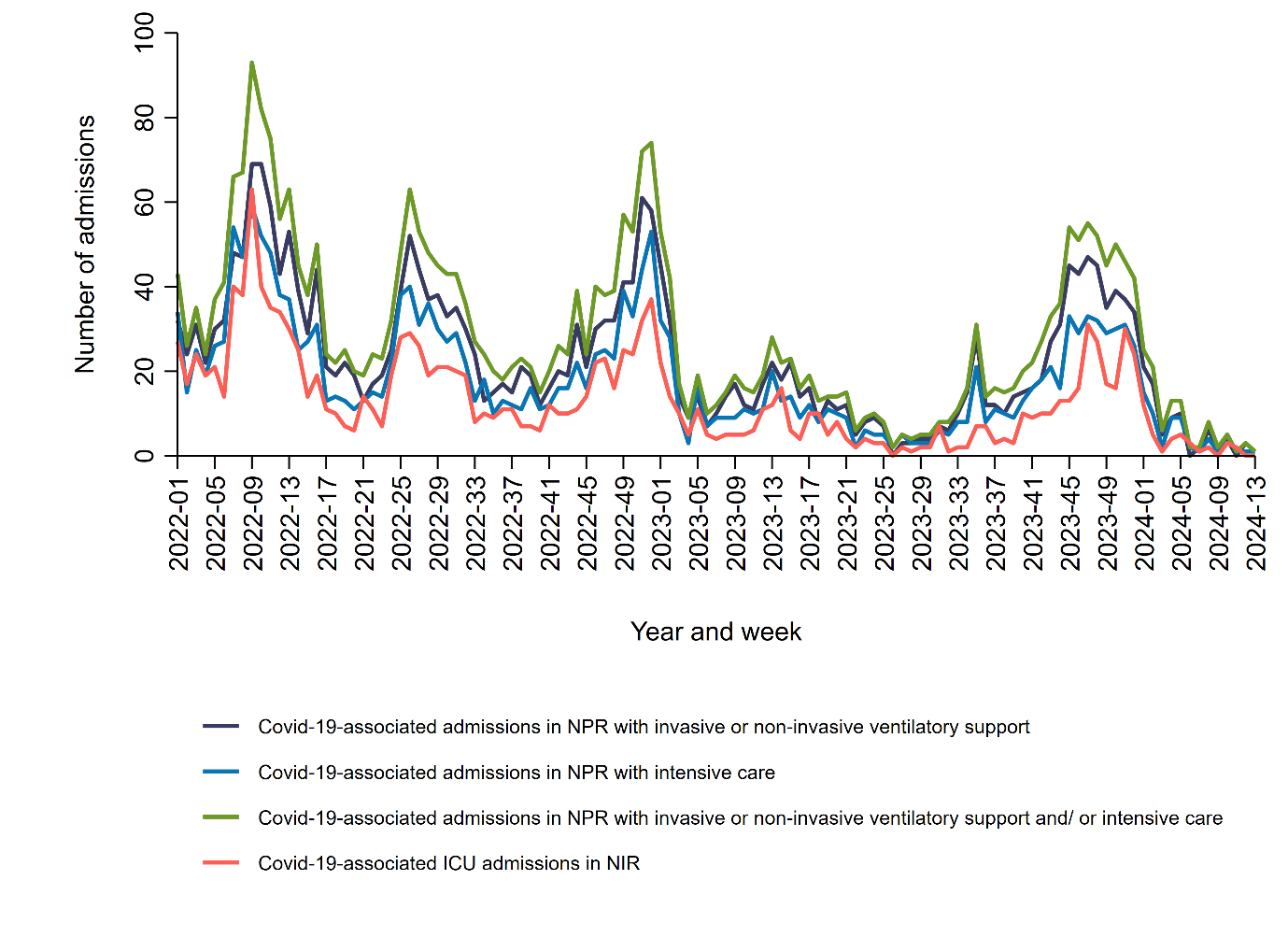


**Figure S8. Weekly number of intensive care unit (ICU) admissions with severe acute respiratory infection (SARI) and positive PCR for SARS-CoV-2 (SARI-COVID) in the surveillance dataset (NPR) by case definition: 1) intensive care (procedure code B0050), 2) invasive or non-invasive ventilatory support (procedure codes GXAV01, GXAV10, GXAV20) and 3) intensive care and/or invasive or non-invasive ventilatory support, compared to ICU admissions with confirmed COVID-19 registered in the Norwegian Intensive Care Registry (NIR), Norway, 03.01.2022 – 31.3.2024**


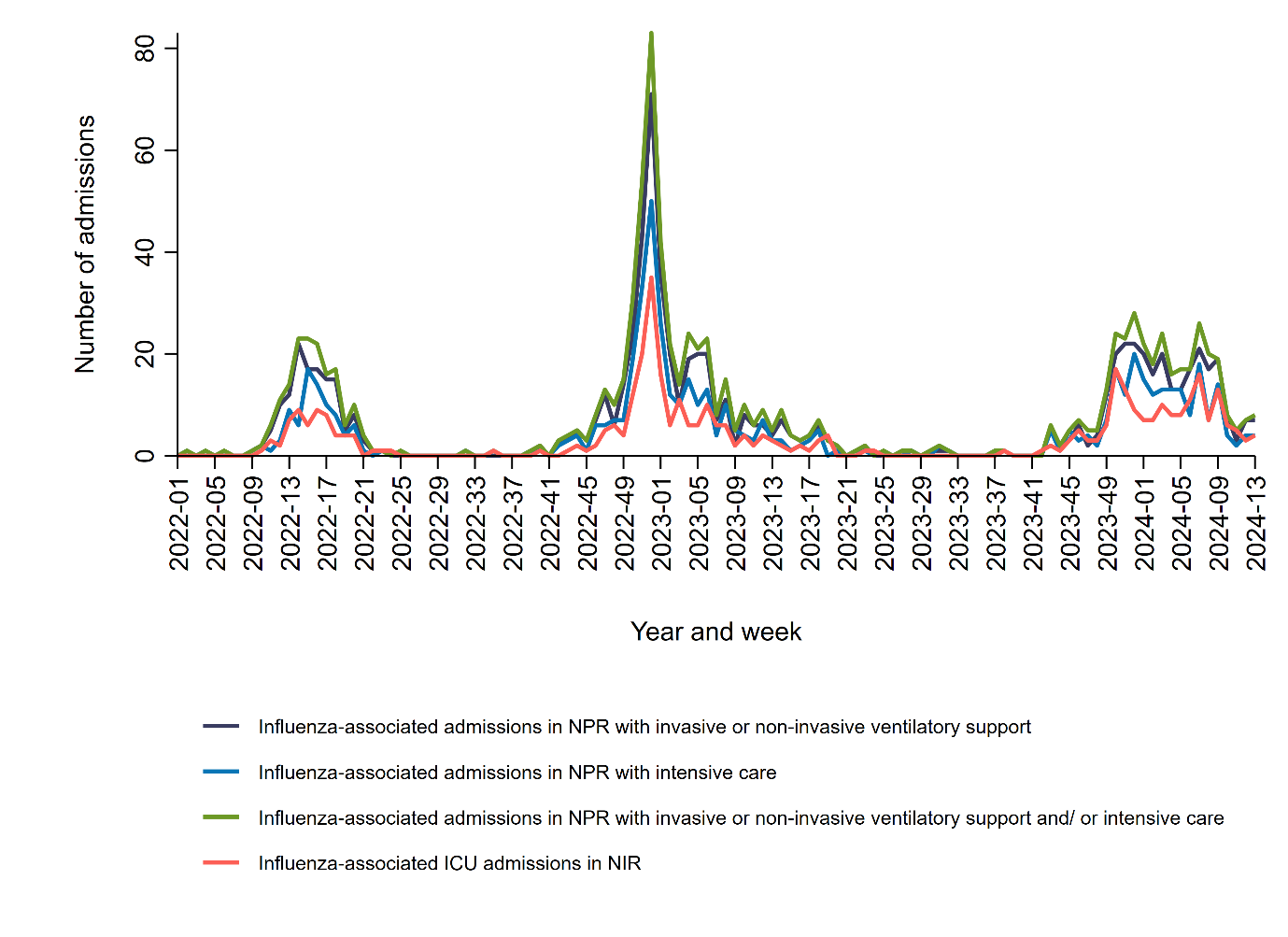


**Figure S9. Weekly number of intensive care unit (ICU) admissions with severe acute respiratory infection (SARI) and positive PCR for influenza virus (SARI- influenza) in the surveillance dataset (NPR) by case definition: 1) intensive care (procedure code B0050), 2) invasive or non-invasive ventilatory support (procedure codes GXAV01, GXAV10, GXAV20) and 3) intensive care and/or invasive or non-invasive ventilatory support, compared to ICU admissions with confirmed influenza registered in the Norwegian Intensive Care Registry (NIR), Norway, 03.01.2022 – 31.3.2024**

To summarize, including IC and/or ventilatory support in the case definition improved the sensitivity but yielded a lower PPV compared to including only IC. Regardless of the choice of procedure codes, it seems difficult to capture the same patient population that is captured by NIR. NIR and the procedure code for IC in NPR seemed to capture partly different patient populations, even though they have the same registration criteria. This may be explained by the fact that in 2022 when the Omicron variant of SARS-CoV-2 dominated in Norway, the number of patients hospitalised with COVID-19 increased, and patients were more widely spread in different departments in the hospitals than earlier during the pandemic. This may have affected coding practices, potentially leading to partly erroneous use of the procedure code for IC, while the coverage of NIR decreased. Another reason for this could be that not all pediatric ICUs report to NIR, thus NPR can be expected to capture more cases in the youngest age groups. Furthermore, the use of the procedure code for IC may not be fully established yet at the hospitals, potentially explaining why some patients were registered in NIR but not in the surveillance dataset as having received IC. It is also possible that some patients registered as “confirmed” in NIR did not have a positive PCR test <=14 days before admissions and <=2 days after discharge, meaning that they would not be defined as SARI-COVID-19 or SARI-influenza cases.

#### Definition of SARI deaths

It is natural for SARI surveillance systems based on manual data collection to define SARI deaths as deaths that occurred in hospital, as identifying deaths shortly after discharge may not be possible. With registry-based surveillance, it can be possible to include also deaths that occurred shortly after discharge, which can be relevant especially for capturing deaths related to complications of SARI. To assess the case definition for deaths associated with SARI, we used the surveillance dataset where only SARI cases with a Norwegian national Id number who died during their hospital stay or within 14 days after discharge were included. We retrieved data on causes of death from the Norwegian Cause of Death Registry and identified deaths where a diagnostic code for SARI (J00-J22, J80, U07.1, U07.2, A37, H65-H67 excluding chronic otitis media) was registered as the underlying or contributing cause of death. We excluded 9 individuals with the same pseudonymized ID. We linked the data on causes of death with the surveillance dataset and excluded cases without a permanent Norwegian ID. We calculated the number and proportion of deaths associated with hospitalisation with SARI where a diagnostic code for SARI was registered as underlying or any cause of death on the death certificate, by timing of death (during hospital stay and ≥1 and ≤14 days after discharge). We also plotted the weekly number of deaths by week of death and case definition (died during hospital stay vs. died during hospital stay or ≤14 days after discharge).

The case definition currently used in the routine surveillance captured a total of 16787 SARI deaths among patients with a permanent Norwegian ID during the study period, of which 12180 (73%) occurred during the hospital stay, and 4607 (27%) 1-14 days after discharge (Table S6, Figure S10). SARI cases who died in-hospital were more likely to have a diagnostic code for SARI registered as underlying or any cause of death compared to SARI cases who died ≥1 and ≤14 days after discharge (Table S6).

**Table S6**. Number and proportion of deaths associated with admission with severe acute respiratory infection (SARI) where a diagnostic code for SARI was registered in the death certificate, by timing of death in relation to the hospital stay, Norway, 28.9.2020 – 31.3.2024

|  |  | **SARI* as underlying cause of death** | | **SARI* as underlying or contributing cause of death** | |
| --- | --- | --- | --- | --- | --- |
| **Timing of death** | **N** | **n** | **%** | **n** | **%** |
| During hospital stay | 12180 | 3408 | 28.0 | 8466 | 69.5 |
| ≥1 and ≤14 days after discharge | 4607 | 876 | 19.0 | 2347 | 50.9 |

*ICD-10 diagnostic codes J00-J22, J80, U071, U072, A37, H65-H67 (acute and unspecified otitis media only)


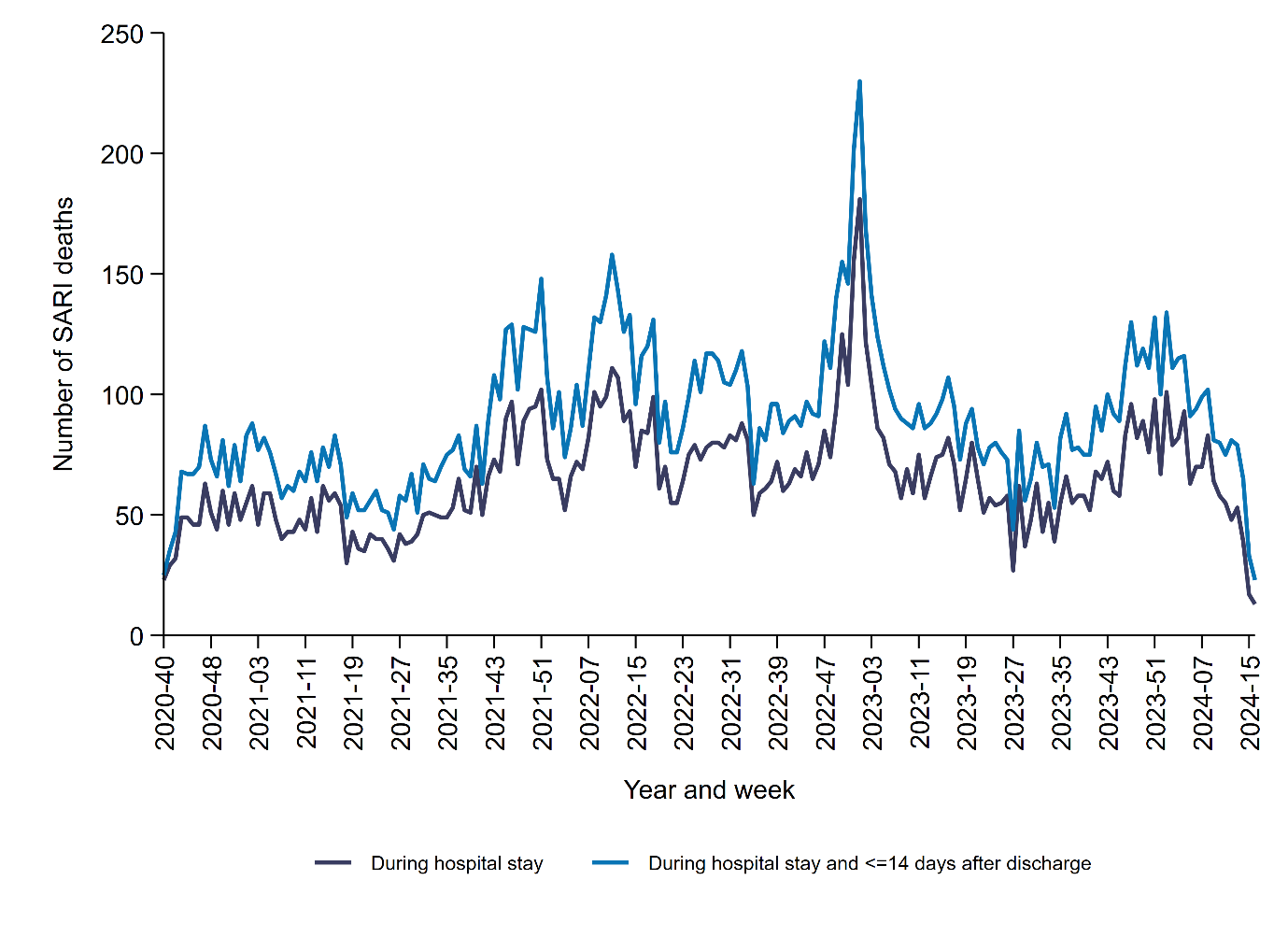


**Figure S10. Weekly number of deaths associated with admission with severe acute respiratory infection (SARI) by case definition for death: 1) in-hospital, 2) in-hospital or ≤14 days after discharge, Norway, 28.9.2020 – 31.3.2024**

*Data for the last weeks of the study period are incomplete.

To summarize, we found that >50% of the SARI cases that died 1-14 days after discharge had SARI as the underlying or contributing cause of death. As cardiac events are a well-known complication of e.g., influenza (11), it could be that even more of the deaths recorded 1-14 days after discharge were related to SARI, even if a diagnostic code for SARI was not registered on the death certificate. If the SARI surveillance system aims to primarily capture deaths due to SARI, including only deaths that occurred in hospital may be warranted.

### **Testing strategy and representativeness of testing**

**Table S7. Number and proportion of admissions with severe acute respiratory infection (SARI) where the patient was tested for SARS-CoV-2, influenza virus and respiratory syncytial virus (RSV) by age group, Norway, 28.9.2020 – 31.3.2024. Only SARI cases with a Norwegian ID are included.**

|  |  | **Tested for SARS-CoV-2** | | **Tested for influenza virus** | | **Tested for RSV** | |
| --- | --- | --- | --- | --- | --- | --- | --- |
| **Age group** | **N** | **n** | **%** | **n** | **%** | **n** | **%** |
| 0-4 years | 23558 | 20426 | 86.7 | 19980 | 84.8 | 18896 | 80.2 |
| 5-14 years | 4894 | 3892 | 79.5 | 3711 | 75.8 | 3374 | 68.9 |
| 15-29 years | 10584 | 8195 | 77.4 | 6918 | 65.4 | 4926 | 46.5 |
| 30-64 years | 46165 | 38429 | 83.2 | 31952 | 69.2 | 22609 | 49.0 |
| 65-79 years | 68395 | 56486 | 82.6 | 50397 | 73.7 | 34457 | 50.4 |
| 80+ years | 59337 | 47818 | 80.6 | 42887 | 72.3 | 27999 | 47.2 |
| Total | 212933 | 175246 | 82.3 | 155845 | 73.2 | 112261 | 52.7 |

**Table S8. Number and proportion of admissions with severe acute respiratory infection (SARI) where the patient was tested for SARS-CoV-2, influenza virus and respiratory syncytial virus (RSV) by county, Norway, 28.9.2020 – 31.3.2024. Only SARI cases with a Norwegian ID are included.**

|  |  | **Tested for SARS-CoV-2** | | **Tested for influenza virus** | | **Tested for RSV** | |
| --- | --- | --- | --- | --- | --- | --- | --- |
| **County** | **N** | **n** | **%** | **n** | **%** | **n** | **%** |
| Agder | 12741 | 7219 | 56.7 | 5703 | 44.8 | 1750 | 13.7 |
| Akershus | 22167 | 18636 | 84.1 | 14138 | 63.8 | 12074 | 54.5 |
| Buskerud | 10079 | 8753 | 86.8 | 7761 | 77.0 | 7667 | 76.1 |
| Finnmark | 3006 | 2116 | 70.4 | 1806 | 60.1 | 1773 | 59.0 |
| Innlandet | 17630 | 15177 | 86.1 | 14062 | 79.8 | 7647 | 43.4 |
| Møre and Romsdal | 10439 | 9004 | 86.3 | 8549 | 81.9 | 3614 | 34.6 |
| Nordland | 10485 | 7932 | 75.7 | 7486 | 71.4 | 7396 | 70.5 |
| Oslo | 21545 | 18308 | 85.0 | 14948 | 69.4 | 10592 | 49.2 |
| Rogaland | 19923 | 16650 | 83.6 | 15607 | 78.3 | 9048 | 45.4 |
| Telemark | 7272 | 6162 | 84.7 | 5198 | 71.5 | 3876 | 53.3 |
| Troms | 5853 | 4890 | 83.5 | 4516 | 77.2 | 4461 | 76.2 |
| Trøndelag | 17334 | 14294 | 82.5 | 13168 | 76.0 | 9155 | 52.8 |
| Vestfold | 11006 | 10008 | 90.9 | 9370 | 85.1 | 7774 | 70.6 |
| Vestland | 30021 | 25281 | 84.2 | 24182 | 80.6 | 18881 | 62.9 |
| Østfold | 12769 | 10271 | 80.4 | 8919 | 69.8 | 6214 | 48.7 |
| Unknown | 663 | 545 | 82.2 | 432 | 65.2 | 339 | 51.1 |
| Total | 212933 | 175246 | 82.3 | 155845 | 73.2 | 112261 | 52.7 |

## **Nowcasting**

To correct for reporting delays, we implemented a simplified nowcasting approach based on (12). We simplified the calculation by first estimating the delay distribution after correcting for the right-truncation (13) using the flexsurv (14) package. We then used the estimated delay distribution to estimate the number of expected admissions when all the data had been reported using a negative-binomial observation model. The model was implemented in the Stan probabilistic programming language (15) using the RStan interface (16).

While not relevant in the retrospective analysis of the nowcasting performance, it was crucial in the real-time nowcasting analysis to take into account that the definitions of SARI-COVID, SARI-influenza and SARI-RSV also included cases that might be removed. From the data we estimated the size of this effect and found that 7.6% of SARI-COVID cases, 5.5% of SARI-influenza cases and 10% of SARI-RSV cases that are registered, are removed. In the implementation we sampled from the posterior of the nowcasting and then for each sample we removed cases by sampling from a binomial distribution based on the probabilities for each case definition. For SARI cases we did not have this effect, as the case definition was based on diagnostic codes alone.

To evaluate how well the nowcasting could predict the final number of admissions, we compared historical nowcasts with the observed data after 5 weeks in Figure S11. The figure shows that nowcasting is important for all the case definitions, but especially important for SARI cases. For the case definitions with laboratory confirmation, delay was shorter and therefore the correction smaller. The correction from nowcasting was still important, especially when estimating the trend in admissions in real-time.


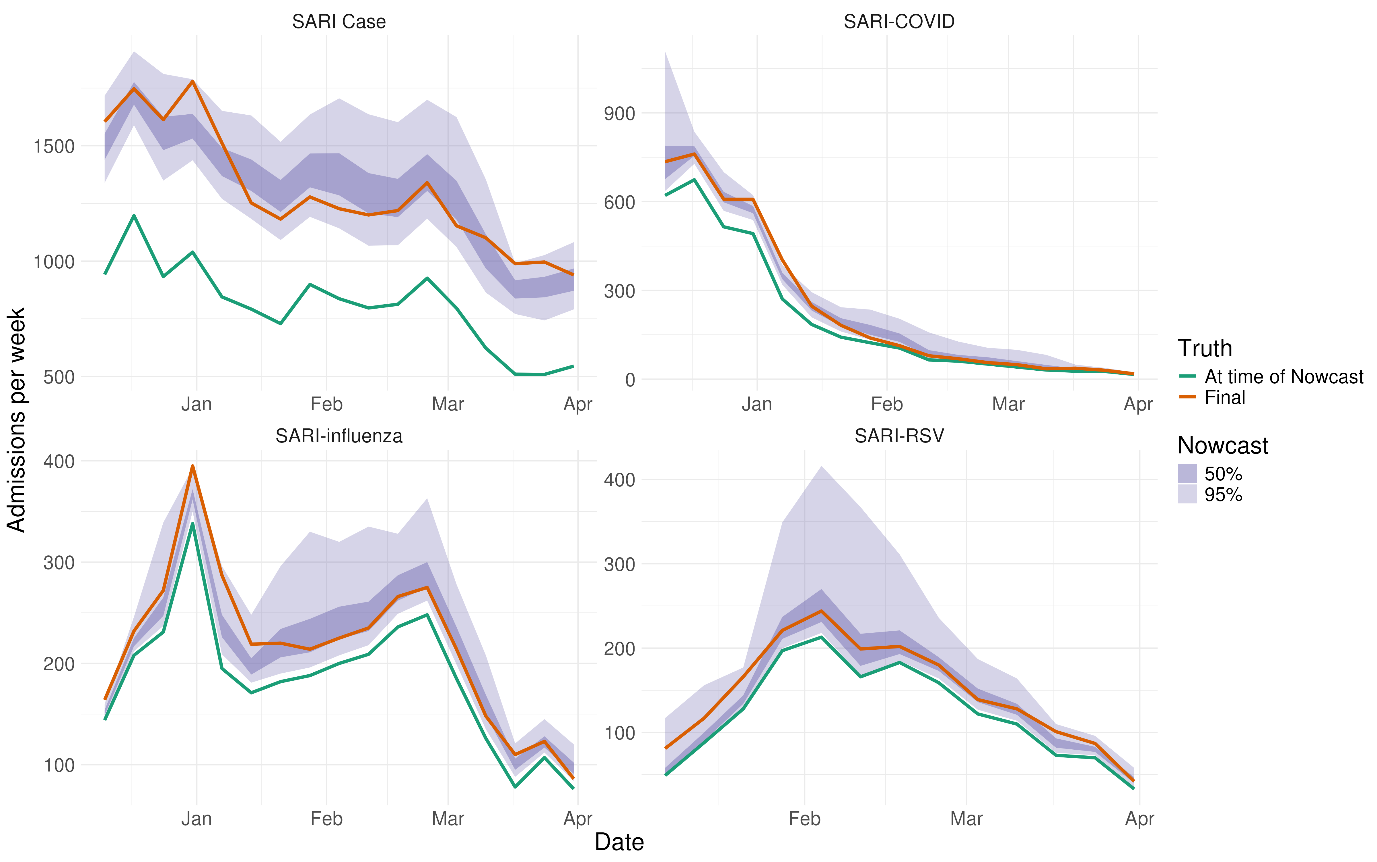
**Figure S11. Nowcasted weekly admissions with severe acute respiratory infection (SARI), SARI-COVID-19, SARI-influenza and SARI-RSV with 50% and 95% prediction intervals, Norway, 01.12.2023 – 01.04.2024. The green line shows the number of reported admissions at the time the nowcasting was performed and the orange line shows the final number of admissions after 5 weeks.**

Overall, the nowcasting provides a prediction with coverage of the final observed number of admissions with only a few weeks where the observed value ended up outside the 95% prediction intervals. For SARI-RSV and partly for SARI-influenza the prediction intervals are quite wide and might overestimate the uncertainty in the nowcasting.

For the nowcasting to be a more a valuable tool in real-time surveillance it is crucial to keep track of reporting dates in all data sources such that proper delay distributions can be estimated. Having a long historical record of the delay distribution would allow estimating changes during the year and for holidays which in our experience would have been important to ensure useful estimates.

## **References**

1. Statistics Norway. StatBank Norway 2025 [Available from: <https://www.ssb.no/en/statbank/>.

2. Roosa Tikkanen RO, Elias Mossialos, Ana Djordjevic, George A. Wharton. International Health Care System Profiles: Norway 2020 [Available from: <https://www.commonwealthfund.org/international-health-policy-center/countries/norway>.

3. Norwegian Medical Products Agency. The Norwegian health care system and pharmaceutical system [Available from: <https://www.dmp.no/en/about-us/the-norwegian-health-care-system-and-pharmaceutical-system>.

4. Cauchi JP, Borg ML, Džiugytė A, Attard J, Melillo T, Zahra G, et al. Digitalizing and Upgrading Severe Acute Respiratory Infections Surveillance in Malta: System Development. JMIR Public Health Surveill. 2022;8(12):e37669.

5. Torres AR, Gómez V, Kislaya I, Rodrigues AP, Fernandes Tavares M, Pereira AC, et al. Monitoring COVID-19 and Influenza: The Added Value of a Severe Acute Respiratory Infection Surveillance System in Portugal. Can J Infect Dis Med Microbiol. 2023;2023:6590011.

6. Instituto de Salud Carlos III. Protocolo para la vigilancia centinela de infección respiratoria aguda grave (IRAG) en hospitales. España Temporada 2023-24. 2023.

7. Norwegian Directorate of Health. Innsatsstyrt finansiering (ISF) – regelverk 2024 [Available from: <https://www.helsedirektoratet.no/tema/finansiering/innsatsstyrt-finansiering-og-drg-systemet/innsatsstyrt-finansiering-isf>.

8. Moustsen-Helms IR, Bager P, Larsen TG, Møller FT, Vestergaard LS, Rasmussen M, et al. Relative vaccine protection, disease severity, and symptoms associated with the SARS-CoV-2 omicron subvariant BA.2.86 and descendant JN.1 in Denmark: a nationwide observational study. The Lancet Infectious diseases. 2024.

9. Seppälä E, Dahl J, Veneti L, Rydland KM, Klüwer B, Rohringer A, et al. Covid-19 and influenza vaccine effectiveness against associated hospital admission and death among individuals over 65 years in Norway: A population-based cohort study, 3 October 2022 to 20 June 2023. Vaccine. 2024;42(3):620-8.

10. Helse Bergen. Norsk Intensivregister 2024 [Available from: <https://www.helse-bergen.no/norsk-intensivregister-nir>.

11. Skaarup KG, Modin D, Nielsen L, Jensen JUS, Biering-Sørensen T. Influenza and cardiovascular disease pathophysiology: strings attached. Eur Heart J Suppl. 2023;25(Suppl A):A5-a11.

12. Günther F, Bender A, Katz K, Küchenhoff H, Höhle M. Nowcasting the COVID-19 pandemic in Bavaria. Biometrical Journal. 2021;63(3):490-502.

13. Lynden-Bell D. A Method of Allowing for Known Observational Selection in Small Samples Applied to 3CR Quasars. Monthly Notices of the Royal Astronomical Society. 1971;155(1):95-118.

14. Jackson C. flexsurv: A Platform for Parametric Survival Modeling in R. Journal of Statistical Software. 2016;70(8):1 - 33.

15. Stan Development Team. Stan Modelling Language Users Guide and Reference Manual. 2.34 ed.

16. Stan Development Team. RStan: the R interface to Stan, R package version 2.32.6. 2024.
